# Prevention of carcinogen-induced oral cancers by polymeric black tea polyphenols via modulation of EGFR-Akt-mTOR pathway

**DOI:** 10.1038/s41598-022-18680-0

**Published:** 2022-08-25

**Authors:** Vaishnavi K. Nimbalkar, Jeet Gangar, Saptarsi Shai, Pallavi Rane, Subham Kumar Mohanta, Sadhana Kannan, Arvind Ingle, Neha Mittal, Swapnil Rane, Manoj B. Mahimkar

**Affiliations:** 1 Mahimkar Lab, Cancer Research Institute (CRI), Advanced Centre for Treatment, Research and Education in Cancer (ACTREC), Tata Memorial Centre (TMC), Kharghar, Navi Mumbai, 410 210 India; 2 Homi Bhabha National Institute, Training school complex, Anushakti Nagar, Mumbai, 400085 India; 3 Clinical Research Secretariat, Advanced Centre for Treatment, Research and Education in Cancer, Tata Memorial Centre, Navi Mumbai, Maharashtra India; 4 Laboratory Animal Facility, Advanced Centre for Treatment, Research and Education in Cancer (ACTREC), Tata Memorial Centre (TMC), Kharghar, Navi Mumbai, India; 5 Department of Pathology, Tata Memorial Hospital, Tata Memorial Centre (TMC), Parel, Mumbai, India; 6 Advanced Centre for Treatment, Research and Education in Cancer (ACTREC), Tata Memorial Centre (TMC), Kharghar, Navi Mumbai, India

**Keywords:** Cancer, Molecular biology, Plant sciences, Health care

## Abstract

The overexpression of Epidermal Growth Factor Receptor (EGFR) and dysregulation of its downstream effector pathways are important molecular hallmarks of oral cancers. Present study investigates the chemopreventive potential of polymeric black tea polyphenols (PBPs)/thearubigins (TRs) in the hamster model of oral carcinogenesis as well as determine the effect of PBPs on EGFR and the molecular players in the EGFR pathway. In dose-dependent manner, pre and concurrent treatment with PBPs (1.5%, 5%, 10%) decreased the number and volume of macroscopic tumors as well as the number and area of microscopic lesions. Interestingly, at 10% dose of PBPs, no macroscopic or microscopic tumors were observed. We observed PBPs mediated dose-dependent decrease in oxidative DNA damage (8OHdG); inflammation (COX-2); proliferation (PCNA, Cyclin D1); expression of EGFR, and its downstream signaling kinases (pAkt, Akt, and mTOR); hypoxia (HIF1α) and angiogenesis (VEGF). There was also a PBPs mediated dose-dependent increase in apoptosis (Bax). Thus, our data clearly indicate that the observed chemopreventive potential of PBPs was due to modulation in the EGFR pathway associated with cell proliferation, hypoxia, and angiogenesis. Taken together, our results demonstrate preclinical chemopreventive efficacy of PBPs and give an insight into its mechanistic role in the chemoprevention of experimental oral cancer.

## Introduction

Tobacco chewing is one of the most important causes of oral squamous cell carcinoma (OSCC). Primary prevention strategies such as reducing exposure to tobacco are impressive however, the addictive nature of nicotine makes them difficult to achieve. In 2019, seven countries had more than 10% prevalences of chewing tobacco among people aged 15–19 years and it is continuing to rise. The habit of chewing tobacco among adolescents is high which leads to chronic exposure to tobacco^1,2^. Thus, it is necessary to target prevention efforts among young tobacco users and reduce healthcare costs in the future^1,2^. Primary chemoprevention is an attractive approach to reduce the risk of disease development^3,4^. The potential of different natural plant-derived compounds against oral cancer is reported in the literature. Phytochemicals like β-carotene, curcumin, green tea, etc. have proven successful in phase I/II clinical trials^5^. Few agents like retinoids, IFNα, and α-tocopherol have reached phase III trials however their toxicity-related issues are pertinent^5–7^. Hence, there is an unmet need for an effective and well-tolerated chemopreventive agent against oral cancer.

Several preclinical studies conducted so far have confirmed the chemopreventive effects of green tea or Epigallocatechin gallate (EGCG) on oral cancer and their molecular targets are known^8–10^. Further, clinical trials have also confirmed their efficacy on patients of leukoplakia as well as HNSCC^11–13^. Black tea (*Camellia sinensis*, family Theaceae) is a commonly consumed beverage in the world, particularly in Asia^14^. During the manufacturing of black tea, a major proportion of monomeric free catechins in the fresh green tea leaf undergo polyphenol oxidase -catalyzed oxidative polymerization process called fermentation, to form oligomers-theaflavins (TFs) and polymers– polymeric black tea polyphenols (PBPs)/ thearubigins (TRs)^15^. PBPs are structurally and chemically ill-defined heterogeneous polymers of flavan-3-ols and flavano-3-ol gallates with di- and tri-benzo tropolone skeletons^16^. Despite being a major polyphenolic component (> 40%) studies on PBPs are limited^17^. The chemopreventive efficacy of PBPs against experimental skin, lung, and colon carcinogenesis is well established^17–20^. However, the chemopreventive potential of PBPs against experimental oral carcinogenesis has not been explored to date. Further dose-dependent chemopreventive potential of PBPs against oral carcinogenesis is least understood which was the rationale to initiate this study.

Epidermal Growth Factor Receptor (EGFR) is a 170 kDa surface protein that is overexpressed in oral pre-cancer and cancer. Its overexpression is related to the more aggressive malignant phenotype, inhibition of cellular apoptosis, and more migratory potential^21^. In preclinical studies molecular targeting of EGFR is a proven successful strategy against oral cancer^22^, However, in clinics, it is less efficient because of poor tolerability in humans^23^. Thus, there is a tremendous need for a highly effective and well-tolerated chemopreventive agent against oral cancer which can modulate the EGFR pathway. There are reports wherein green tea is observed to downregulate overexpression of EGFR in head and neck squamous cell carcinoma (HNSCC) cell lines^24^. However, there is no report to date on the effect of black tea on EGFR and its downstream signaling targets in an in vitro or in vivo model. In the present study, our objective was to evaluate the chemopreventive potential of different doses of PBPs in experimental oral cancer using hamster buccal pouch (HBP) as a model system and to understand the underlying molecular mechanism. We have analyzed the chemopreventive potential of PBPs on key cellular processes driving oral carcinogenesis such as oxidative DNA damage, inflammation, proliferation, and apoptosis. We have also studied the effect of PBPs on EGFR and its downstream targets, hypoxia, and angiogenesis.

## Materials and Methods

### Materials

7, 12-dimethylbenz(a)anthracene (DMBA) (purity ~ 95%) was purchased from Sigma-Aldrich Chemical Company (St. Louis, MO, USA). Antibodies for PCNA, Cox-2, cyclinD1, Bax, Bcl-2, VEGF, 8-OHdG were purchased from Abcam (Cambridge, MA, USA). Antibody for Actin was purchased from Santa Cruz Biotechnology (Santa Cruz, CA, USA). Antibodies for EGFR, Akt, pAkt, and mTOR were procured from Cell Signalling Technology (Beverly, MA, USA). Antibody for HIF1 α was purchased from Sigma-Aldrich (St. Louis, MO, USA). Anti- Rabbit, and Anti- Mice IgG biotinylated secondary antibodies were procured from Vector laboratories (Burlingame, CA, USA). The anti-rabbit HRP conjugated secondary antibody was purchased from GE Healthcare (Chicago, Illinois, US). Dako EnVision FLEX kit for VEGF immunohistochemical (IHC) analysis was purchased from Agilent Technologies (Santa Clara, CA, USA). Clarity western ECL substrate for western blot visualization was purchased from Bio-Rad Laboratories, Inc. (Hercules, CA, USA). Western Bright ECL kit for western blot visualization was purchased from advansta Inc. (San Jose, CA, USA).

### Methods

#### Preparation of PBPs extract from black tea and analysis of PBPs

A popular brand of black tea (Red Label, Brooke bond, Mumbai, India) was used to prepare polymeric black tea polyphenols (PBPs) extract. Five fractions of PBPs were extracted using the Soxhlet apparatus (Borosil Glass Works Ltd., Mumbai, India) and sequential use of different organic solvents as per established protocol^25^. The details are given in (Supplementary text 1. I). 1.5%, 3%, 5%, and 10% PBP doses were fed to animals after confirming their purity using previously established thin layer chromatography and MALDI-TOF techniques^17^. MALDI- TOF analysis details are given in (Supplementary text 1. II). Further UV absorption spectra of each dose were analyzed to ascertain their purity and dose-related yield^17^. MALDI-TOF analysis was used to confirm that PBPs are free from other potentially biologically active components in tea as caffeine, monomeric black tea polyphenols (EGCG) and oligomeric black tea polyphenols (theaflavins) as per established protocol^17^.

#### Animal treatment

**Figure 1:**
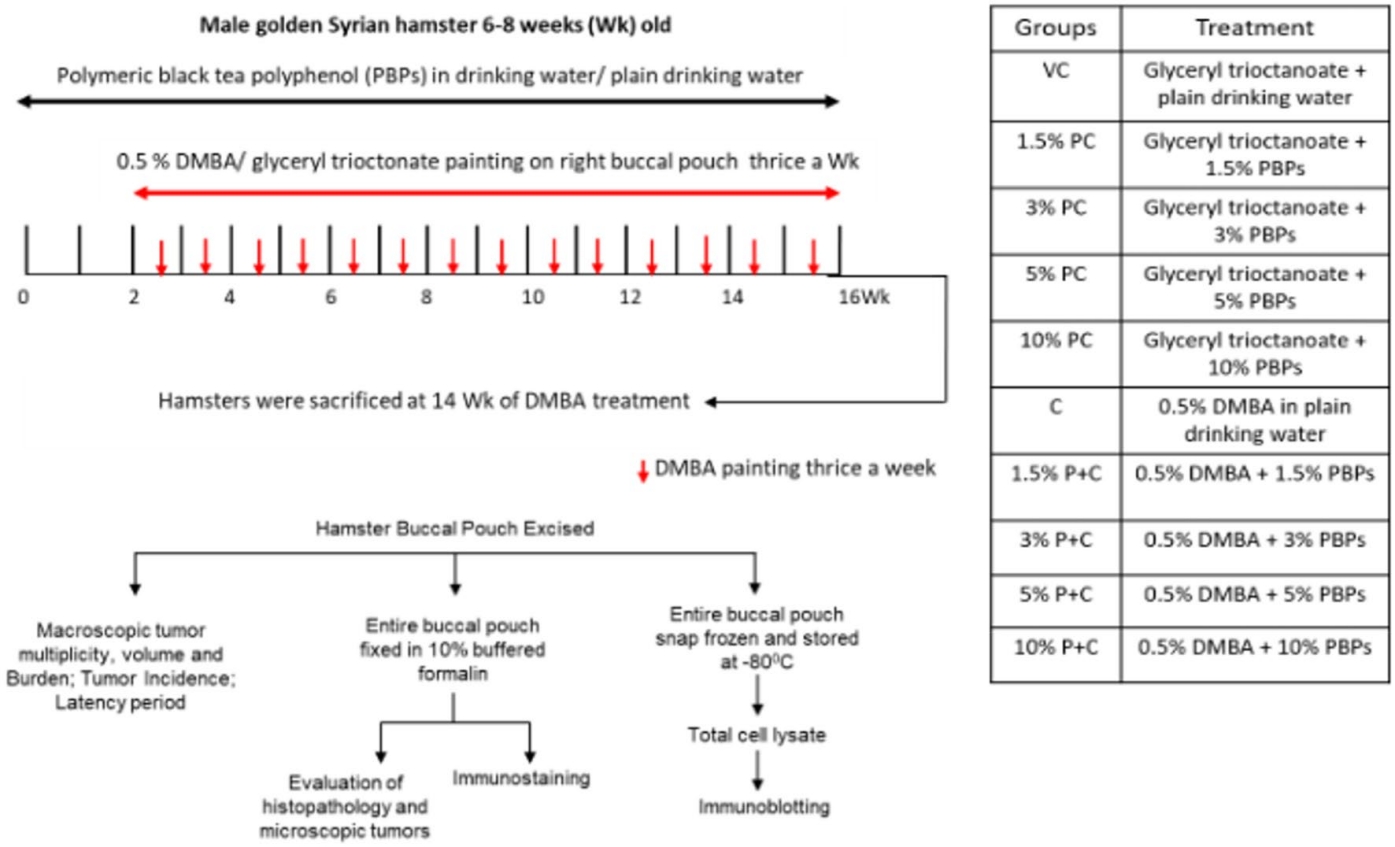
Experimental design for studying the effect of pre and concurrent treatment of different doses of black tea-derived PBPs extract on DMBA induced oral carcinogenesis in the hamster model. 6–8 weeks old male golden Syrian hamsters were randomised into ten groups as vehicle control (VC) PBP control (1.5% PC, 3% PC, 5% PC, 10% PC) carcinogen (C) PBP + carcinogen (1.5% P + C, 3% P + C, 5% P + C, 10% P + C) as shown in tabular format. PBP control (1.5% PC, 3% PC, 5% PC and 10% PC) and PBP + carcinogen (1.5% P + C, 3% P + C, 5% P + C, 10% P + C) group animals received 1.5%, 3%, 5%, 10% PBPs as sole source of drinking water for initial two weeks while vehicle control (VC) and carcinogen (C) group animals received plain drinking water. Further right buccal pouch of animals in carcinogen (C) and PBPs + carcinogen (1.5% P + C, 3% P + C, 5% P + C and 10% P + C) group were topically painted with 0.5% DMBA in glyceryl trioctanoate three times a week for fourteen weeks and were continued on plain drinking water and 1.5%, 3%, 5% and 10% PBPs respectively. Right buccal pouch of animals from vehicle control (VC) and PBP control (1.5% PC, 3% PC, 5% PC, 10% PC) groups were topically applied with glyceryl trioctanoate three times a week for fourteen weeks and were continued on plain drinking water and 1.5%, 3%, 5% and 10% PBPs respectively. Sacrifice was done after fourteen weeks of DMBA treatment. Entire buccal pouch was excised and evaluated for macroscopic tumor multiplicity, volume and burden. Then it was either fixed in 10% buffered formalin or snap frozen and stored at − 80 °C.

All animal studies were conducted after approval from the Institutional Animal Ethics Committee (Sanction no: 14/2017) endorsed by the Committee for Control and Supervision of Experiments on Animals (CPCSEA) Government of India. All methods performed in the study were in accordance with the guidelines and regulations of Institutional Animal Ethics Committee (Sanction no: 14/2017) endorsed by the Committee for Control and Supervision of Experiments on Animals (CPCSEA) Government of India. Inbred male Syrian hamsters (6–8 weeks old; Animal house, ACTREC, India) were randomized and housed under standard conditions: 22 ± 2 °C, 45% ± 10% relative humidity, and a 12-h light/dark cycle. Animals received a standard pellet diet ad libitum (Diet composition in (Supplementary text 2. I) and (Supplementary Table 3). All the hamsters in the study were euthanized by CO_2_ inhalation in CO_2_ chamber. The detailed animal treatment protocol is provided in Fig. 1 and (Supplementary text 2. II). Hamsters were sacrificed after 14 weeks of carcinogen treatment and both right, as well as a left buccal pouch, were evaluated for macroscopic tumor parameters such as tumor multiplicity (average no of tumors per animal) volume, and burden by veterinarian blinded for treatment groups. Macroscopic buccal pouch tumors (of size > 1 mm) were counted by a pathologist blinded for treatment groups. Maximum and minimum diameters of the tumor were measured using a digital vernier caliper to calculate tumor volume and burden as per established protocol (Supplementary text 2. IV). After evaluation, the entire buccal pouch tissue was either fixed in 10% buffered formalin or snap-frozen and stored at  − 80 °C. Fixed tissue was used for further histopathological evaluations, microscopic tumor measurement, and IHC. While snap-frozen tissue was further used for western blotting.

#### Evaluation of histopathology and microscopic tumor parameters (number and area) of the buccal pouch

We for the first time evaluated microscopic lesions and areas of HBP as per our best knowledge, while the method is previously established for the evaluation of lung lesions^17^. A similar method has been adopted here the details of which are provided in the (Supplementary text 2. V). The microscopic buccal pouch lesions were categorized into -hyperplasia, dysplasia, and squamous cell carcinoma (SCC)^26^. The average number and area of these microscopic lesions were compared between groups by two independent pathologists blinded for the treatment groups.

#### Protein immunoblotting

The total cell lysate was prepared from snap-frozen entire buccal pouch tissue as per established protocol^27^. The total cell lysate was aliquoted and stored at − 80 °C till use. Lysates were evaluated for their protein content using the Folin-Lowry method of protein estimation^28^. Randomly five animals from each group were evaluated for western blotting. Standardization performed for each molecular marker for its primary antibody, secondary antibody, and quantity of total cell lysate protein used is given in detail (Supplementary Table 7). The detailed protocol for western blotting is provided in (Supplementary text 3). β-actin was used as a loading control protein. The WB images provided in the figure panel are cropped for uniforme presentation of all blots images; however the whole blot uncropped images are provided in the supplementary text. Densitometry analysis of various analyte proteins and respective loading control protein from the same blot was performed using chemi-doc software (Bio-Rad Laboratories). Densitometric readings of analyte proteins were divided by their respective loading control protein from the same blot to calculate relative optical density.

#### Immunohistochemical staining and evaluation

For IHC staining, 5 μm thick sections were mounted on poly-L-lysine coated slides and were processed for IHC evaluation. The details of which are given in (Supplementary text 4). Randomly five animals from each group were evaluated for immunohistochemistry. Standardization details of IHC specific for each molecular marker are provided in Supplementary Table 8. For nuclear staining proteins (8-OHdG, PCNA, and HIF1α) cytoplasmic staining proteins (COX-2, VEGF, Bax, and Bcl2) and membrane staining protein (EGFR) semiquantitative analysis was conducted using ImageJ 1.43 (NIH) software. Percentage positive cells were calculated as the number of positively stained cell*100/ total number of cells in photomicrographs of HBP tissue. At least 10 different randomly selected fields were counted for a minimum of 1000 cells at 400X magnifications. The entire buccal pouch of at least five animals from each group was evaluated. Percentage positive cells from PBPs + carcinogen (1.5%, 5%, and 10%) group were compared with percentage positive cells of carcinogen group. Percentage positive cells of the carcinogen group were compared with percentage positive cells of vehicle control and PBPs control (1.5%, 5%, and 10%) group.

#### Evaluation of cytotoxicity of PBPs

Cytoxicity of PBPs was determined by variety of experimental measures as every week evaluation of weight of hamsters from different treatment groups; analysis of expression of proliferation (PCNA, Cyclin D1) inflammation (Cox2) oxidative DNA damage (8-OHdG) and apoptosis markers (Bax, Bcl2) in hamster buccal pouch tissue from different control groups- Vehicle control and PBPs (1.5%, 5%, 10%) control using immunohistochemical (IHC) and immunoblotting (WB) technique.

#### Power calculation

Power calculation for all the experiments regarding the tumorigenicity and for measuring the target protein markers through IHC and western blotting data was carried out using PASS v2019. One-way Analysis of Variance (F-test) was used to calculate the power of 4 experimental groups (Carcinogen, 1.5%PBPs + C, 5%PBPs + C, and 10%PBPs + C) for the number of tumors in the animal experiment and each of the biomarkers of IHCs and western blotting. The sample size varied for the number of tumors in the animal study while it was 20 with 5 in each combination group for IHC and western blotting. The standard deviation of the group means and within-group standard deviation was used to estimate the effect sizes and to finally empower our study while achieving the desired power of > 80% at a 0.05 level of the significance level.

#### Statistical analysis

Shapiro–Wilk test was carried out before each of the analyses to check the normal distribution between the variables. Increase from initial to final body weight, microscopic tumor number and area comparison, mean of each analyte protein with its respective loading controls in western blotting, and evaluation of IHC stained slides were compared between different experimental groups using ANOVA followed by Bonferroni correction or a Kruskal Wallis test. Macroscopic tumor number, volume, and burden between each experimental group were compared using the non-parametric Mann Whitney U-test. Additionally, Pearson (r) or Spearman (rho) correlation matrix between macroscopic and microscopic tumor numbers, all the biomarkers of IHCs and western blots, and Spearman's rho correlation coefficient were calculated. Normally distributed data were represented with mean ± SD while not-normal data was represented with median interquartile range (IQR). A P-value of less than 0.05 was considered to be statistically significant. All the analyses were performed using IBM SPSS 25.0.

### ARRIVE guidelines statement

The study reported in the manuscript is in accordance with ARRIVE guidelines.

## Results

### Evaluation of purity of PBPs extract

To ascertain that each extracted batch of PBPs is free from other components of black tea i.e. caffeine, EGCG (monomeric black tea polyphenols) and theaflavins (oligomeric black tea polyphenols) they were analyzed by TLC and MALDI- TOF technique (Supplementary text 1. II) similar to a previously published report from our lab (Supplementary Fig. 1)^17^. Extracted PBPs were shown to be free from known biologically active components like caffeine, EGCG, theaflavin, and thus observed biological activity is attributed to only PBPs.

### Analysis of macroscopic buccal pouch tumors and microscopic buccal pouch lesions

#### PBPs decreased macroscopic tumor multiplicity, volume, and burden

Animals from vehicle and 1.5%, 3%, 5%, 10% PBPs control group showed no visible tumor. However, 100% tumor incidence was observed in Carcinogen and 1.5% PBPs + carcinogen group; 75% tumor incidence in 5% PBPs + carcinogen group and no visible tumor observed in any animal from 10% PBPs + carcinogen group. Treatment of PBPs showed trend positive dose-dependent significant decrease in average buccal tumor numbers in 1.5% PBPs + carcinogen, 5% PBPs + carcinogen, and 10% PBPs + carcinogen group as compared to carcinogen group (Table 1). There was no significant difference between tumor multiplicity of group 3% PBPs + carcinogen and 5% PBPs + carcinogen; hence for all further biomolecular analysis, only (1.5%, 5%, and 10%) PBPs + carcinogen tissues were used. Average tumor volume was highest in carcinogen group which goes on decreasing in dose dependant manner with trend positive in (1.5%, 5%, and 10%) PBPs + carcinogen group (Table 1). However, there was no significant difference between tumor volume of groups 1.5% PBPs + carcinogen and 3% PBPs + carcinogen also 3% PBPs + carcinogen and 5% PBPs + carcinogen. Tumor burden goes on decreasing with increasing exposure to PBPs in a dose-dependent manner with trend positive in (1.5%, 5%, and 10%) PBPs + carcinogen group (Table 1). However, there was no significant difference between tumor burden of groups 3% PBPs + carcinogen and 5% PBPs + carcinogen.

**Table 1:** Effect of pre and concurrent treatment of PBPs on DMBA induced hamster buccal pouch carcinogenesis.

Groups	*N*	Body weight (g)		Hamster buccal pouch tumors			*P*-value
		Initial^®^	Final^®^	Multiplicity^®^	Volume^®^ (mm^3^)	Burden^®^ (mm^3^)	
VC	31	75.22 ± 10.41	113.59 ± 13.25	0	0	0	
1.5% PC	18	72.52 ± 11.79	111.11 ± 16.97	0	0	0	
3% PC	13	76.45 ± 7.92	115.18 ± 11.59	0	0	0	
5% PC	14	74.34 ± 07.97	111.41 ± 21.59	0	0	0	
10% PC	14	76.79 ± 08.90	117.26 ± 16.59	0	0	0	
C	37	76.65 ± 08.65	114.08 ± 12.51	5.64 ± 1.36	7.33 ± 4.04	39.77 ± 19.35	
1.5% P + C	19	72.02 ± 10.84	105.89 ± 21.58	2.58 ± 1.02*	1.31 ± 0.63*	3.62 ± 2.50*	≤ 0.0001
3% P + C	21	72.95 ± 12.87	112.62 ± 18.37	1.71 ± 0.96*^†^	1.12 ± 0.53*	1.97 ± 1.26*^†^	≤ 0.0001
5% P + C	22	74.45 ± 08.75	108.70 ± 14.06	1.23 ± 1.07*^†^	1.06 ± 0.97*^†^	1.89 ± 1.91*^†^	≤ 0.0001
10%P + C	22	78.15 ± 10.20	123.03 ± 14.44	0.00 ± 0.00*^†‡^	0.00 ± 0.00*^†‡^	0.00 ± 0.00*^†‡^	≤ 0.0001

*Significantly different from Carcinogen *P* ≤ 0.0001, † Significantly different from 1.5% PBPs + Carcinogen *P* ≤ 0.0001, ‡ Significantly different from 5% PBPs + Carcinogen *P* ≤ 0.0001, ® Represented as Mean ± standard deviation.

6–8 weeks old hamsters were randomised into ten groups as follows: Vehicle control (VC) 1.5% PBPs control (1.5% PC) 3% PBPs control (3% PC) 5% PBPs control (5% PC) 10% PBPs control (10% PC) Carcinogen (C) 1.5% PBPs + Carcinogen (1.5% P + C) 3% PBPs + Carcinogen (3% P + C) 5% PBPs + Carcinogen (5% P + C) 10% PBPs + Carcinogen (10% P + C). PC (1.5%, 3%, 5% and 10%) and P + C (1.5%, 3%, 5% and 10%) were primed with 1.5%, 3%, 5% and 10% PBPs respectively for two weeks as a sole source of drinking water. VC and C groups were given plain drinking water for two weeks. After two weeks of priming right buccal pouches of animals of C and P + C (1.5%, 3%, 5% and 10%) groups were topically applied with carcinogen i.e., 0.5% DMBA dissolved in glyceryl trioctanoate for three times a week and such fourteen weeks. Animals of PC (1.5%, 3%, 5% and 10%) and P + C (1.5%, 3%, 5% and 10%) groups were given 1.5%, 3%, 5% and 10% PBPs respectively through drinking water, while those of VC and C groups were given plain drinking water for fourteen weeks. All animals were sacrificed after sixteen weeks. Initial and final body weights are represented as mean ± standard deviation. Macroscopic tumor multiplicity, volume and burden were calculated of all animals in each sacrifice and were compared between the groups (*^, †, ‡^*P*-value ≤ 0.0001, Mann–Whitney Wilcoxon test).

#### PBPs decreased the number of microscopic lesions

Tile scan images of representative HBP H&E-stained sections from different treatment groups are shown in Supplementary Fig. 5. The buccal pouch epithelium of animals from the vehicle control group and 1.5%, 5%, 10% PBPs control group was unaltered. After 14 weeks of carcinogen treatment, all the treatment groups animals showed the presence of microscopic SCC except animals from the 10% PBPs + carcinogen group. The average number of hyperplasia, dysplasia and SCC lesions showed significant reduction across groups 1.5% PBPs + carcinogen, 5% PBPs + carcinogen, and 10% PBPs + carcinogen with the increasing exposure of PBPs (Table 2). Furthermore, when the macroscopic tumor count of 3 independent animals each from all groups was compared with the microscopic tumor count of the same animals by correlation analysis it was found to be highly correlated (*p* < 0.001) with correlation coefficient R = 0.962 (Supplementary Table 5, Supplementary Fig. 7A).

**Table 2:** Effect of pre and concurrent treatment of PBPs on microscopic hamster buccal pouch tumor multiplicity and area.

Group (*n* = 3)	Hyperplasia	*p*-value	Dysplasia	*p*-value	Squamous cell carcinoma	*p*-value
Carcinogen	6.57 ± 0.25		4.26 ± 0.13		4.30 ± 0.18	
1.5% P + C	5.10 ± 0.06	**0.016**	3.30 ± 0.04	**0.015**	2.09 ± 0.07	**0.015**
5% P + C	4.21 ± 0.22		2.10 ± 0.05		1.37 ± 0.49	
10% P + C	2.91 ± 0.22		0.52 ± 0.19		0.00 ± 0.00	

Represented as Mean ± standard deviation, Kruskal–Wallis test applied.

The number of each proliferative lesion assigned by pathologists were counted in each tile scan (50 microns apart). The total number of each proliferative lesion type was counted in every tile scan of each hamster and expressed as an average number of each type of lesion per hamster.

#### PBPs decreased the area of microscopic buccal pouch lesions

The average area of hyperplasia, dysplasia, and SCC lesions showed significant reduction across groups—1.5% PBPs + carcinogen, 5% PBPs + carcinogen, and 10% PBPs + carcinogen also mean area of total lesions in the entire buccal pouch decreased with the increasing exposure of PBPs (Table 3, Supplementary Fig. 6).

**Table 3:** Effect of pre and concurrent treatment of PBPs on microscopic hamster buccal pouch tumor multiplicity and area.

Group (*n* = 3)	Hyperplasia (μm^2^)	*p*-value	Dysplasia (μm^2^)	*p*-value	Squamous cell carcinoma (μm^2^)	*p*-value
Carcinogen	932,952.26 ± 33,196.02		109,808.65 ± 23,450.64		1,374,203.22 ± 196,799.31	
1.5% P + C	525,361.49 ± 34,813.54	< 0.001	57,714.07 ± 5708.68	< 0.001	446,932.03 ± 27,011.96	< 0.001
5% P + C	350,525.82 ± 39,286.78		14,437.46 ± 1112.44		259,802.41 ± 23,516.15	
10% P + C	142,787.57 ± 15,718.31		5492.83 ± 4764.86		0.00 ± 0.00	

Represented as Mean ± standard deviation, ANOVA test applied.

The area of each proliferative lesion assigned by pathologists were counted in each tile scan (50 microns apart). The areas of lesions were counted by using Image J software in every tile scan for all animals and expressed as the average area of each type of lesion per hamster.

### PBPs administration did not induce any cytotoxicity to hamster buccal pouch tissue

**Figure 2:**
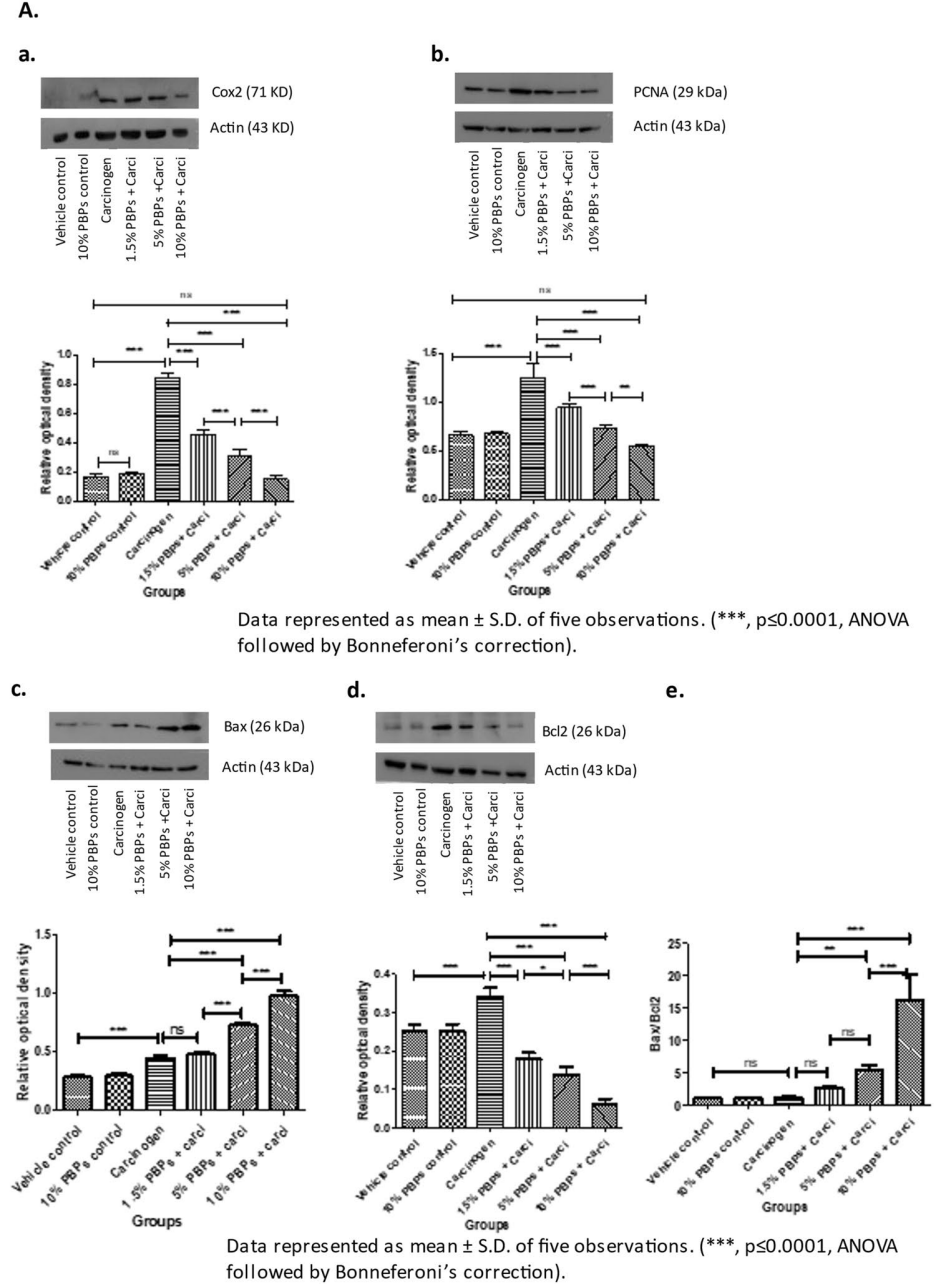

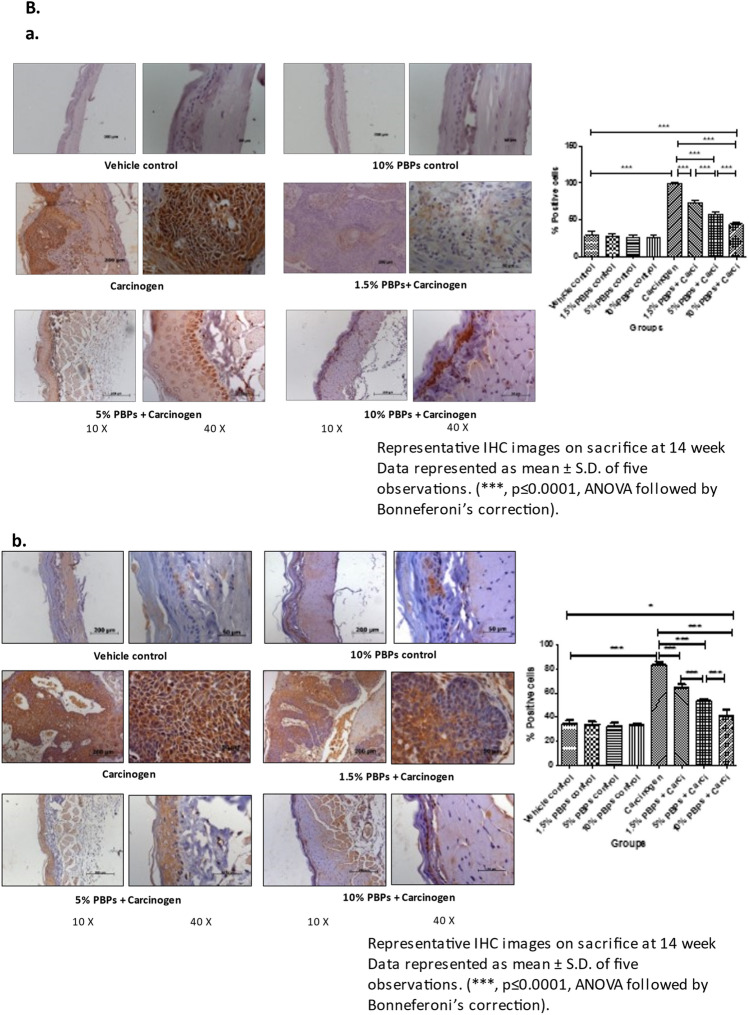

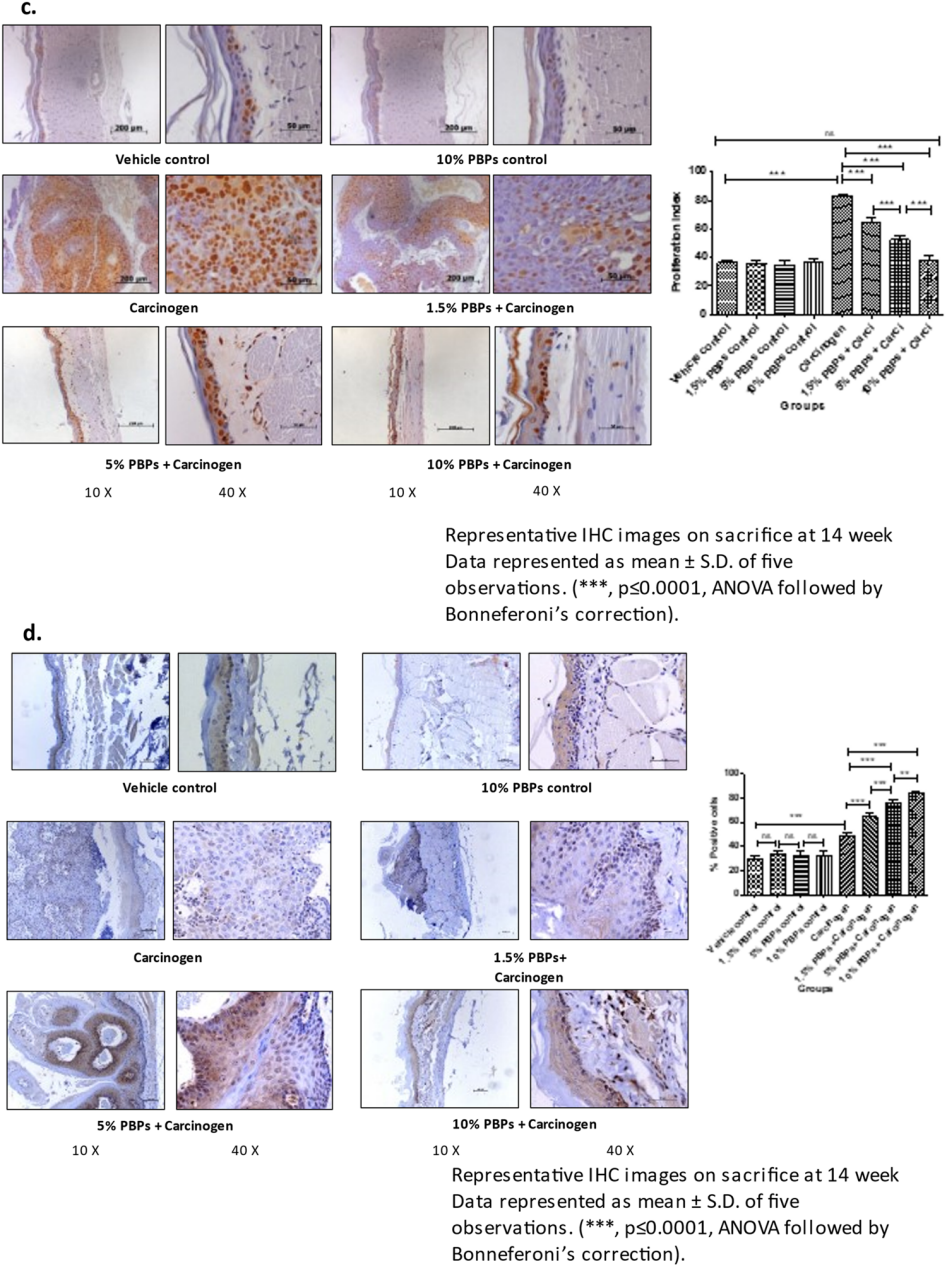

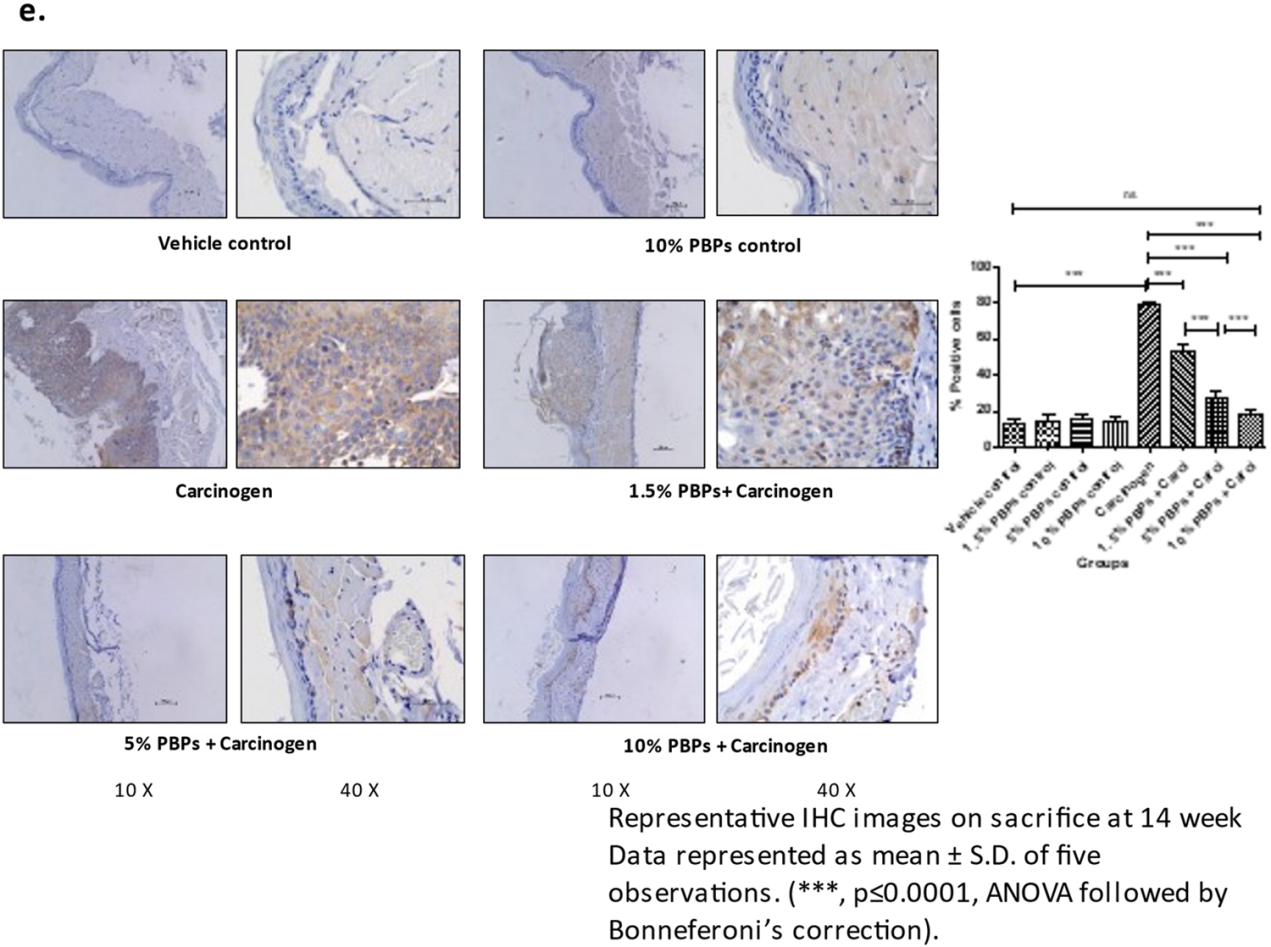
Effect of pre and concurrent treatment of different doses of PBPs on DNA damage, inflammation, proliferation, and apoptosis markers in experimental oral cancer. (**A**) Representative blots and relative densitometric levels of (**a**) Cox2, (**b**) PCNA, (**c**) Bax and (**d**) Bcl2. Data represented as mean ± S.D. of five observations. (***, *p* ≤ 0.0001, ANOVA followed by Bonneferoni’s correction). (**B**) Representative photomicrographs showing immunohistochemical detection of (**a**) 8-OHdG, (**b**) Cox-2 (**c**) PCNA and (**d**) Bax and (**e**) Bcl2. Data represented as mean ± S.D. of five observations. (***, *p* ≤ 0.0001, ANOVA followed by Bonneferoni’s correction).

We did not observed any PBP-mediated cytotoxicity which is evident from variety of experimental measures as follows- throughout the experiment animals drank equal quantity of water and all doses of PBPs i.e. 1.5%, 3%, 5%, 10% PBPs (Supplementary Table 4); there was no moratality to any animal from any treatment group; there was constant increase in body weights of animals from all treatment groups and at the end of the experiment the final body weights of all animals were higher as compared to their initial body weight with no statistically significant difference (Supplementary Fig. 2). Molecular marker analysis using IHC and WB techniques showed that there was no difference in expression levels of proliferation markers—PCNA, CyclinD1; inflammation marker- Cox2; oxidative DNA damage marker- 8-OHdG; apoptosis marker- Bax, Bcl2 between vehicle control and all PBPs (1.5%, 5% and 10%) control groups (Fig. 2B a–e) (Supplementary Fig. 9A, C, D, E and F).

### Pre and concurrent treatment of PBPs decreased carcinogen-induced oxidative DNA damage, inflammation, and proliferation, and increased carcinogen-induced apoptosis

PBPs decrased macroscopic tumor number, volume, burden and microscopic tumor number, area in dose dependent manner which clearly proves their chemopreventive potential in HBP carcinogensis. However the mechanistic aspect behind chemopreventive potential of PBPs is unknown. At 10% PBPs dose we observed no macroscopic and microscopic tumors which interested us to find out of difference in molecular expression of different biomarkers which drive oral carcinogenesis. Limited information is available on molecular mechanism of PBPs which can be attributed to their chemopreventive activity. None study till date has reported effect of PBPs on expression of EGFR and its downstream effector pathway in experimental oral cancer. Thus in the present study we evaluated effect of PBPs on DNA damage, inflammation, proliferation, apoptosis related biomarkers and major downstream signaling pathway EGFR-Akt-mTOR.

#### PBPs decreased carcinogen-induced oxidative DNA damage, inflammation, and proliferation

Expression of 8-OHdG, Cox2, and PCNA was comparable among all the vehicle control and all (1.5%, 5%, and 10%) PBPs control groups. While in the carcinogen group it was significantly higher as compared to the vehicle control. Expression of 8-OHdG, Cox2, and PCNA decreased with the increasing dose of PBPs and the decrease was statistically significant within the groups. 8-OHdG, Cox2, and PCNA expression were minimum in 10% PBPs + carcinogen among all PBPs + carcinogen treatment groups (Fig. 2B a–c). The observed relative differences in Cox2 and PCNA expression by immunohistochemistry agreed with results observed by immunoblotting. Expression of Cox2 and PCNA was low in control groups (Supplementary Fig. 9A and D) which significantly increased in the carcinogen group and further decreased significantly with increased exposure of PBPs (Fig. 2A a and b). Thus, pre and concurrent treatment of PBPs decreased carcinogen-induced high expression of 8-OHdG, Cox2, and PCNA in a dose-dependent manner which suggests their anti-oxidant, anti-inflammatory, and anti-proliferative capacity.

#### PBPs increased carcinogen-induced apoptosis

The expression of Bax was minimum and not significantly different among vehicle control and all (1.5%, 5%, and 10%) PBPs control groups (Supplementary Fig. 9E). Expression of Bax was significantly increased in the carcinogen group. PBP exposure further enhanced carcinogen-induced Bax expression significantly in a dose-dependent manner (Fig. 2A c). Similar to Bax, expression of Bcl2 was minimum and was significantly different in control groups (Supplementary Fig. 9F). Bcl2 expression was highest in the carcinogen group. Bcl-2 expression showed a significant polyphenol mediated decrease in the PBPs + carcinogen group as compared to the carcinogen group (Fig. 2A d). The ratio of Bax/Bcl-2 was similar in-vehicle control, 10% PBPs control, and carcinogen group. Bax/Bcl-2 ratio increased significantly in 5% and 10% PBPs + carcinogen as compared to carcinogen group (Fig. 2A e). Similar to immunoblotting, relative differences in expression of Bax and Bcl2 were further substantiated by their immunohistochemical staining respectively (Fig. 2B d and e). Hence results of IHC and western blotting together confirmed the pro-apoptotic activity of PBPs.

### Pre and concurrent treatment of PBPs modulated carcinogen-induced EGFR pathway

#### PBPs decreased carcinogen-induced expression of proliferation markers- EGFR and Cyclin D1

**Figure 3:**
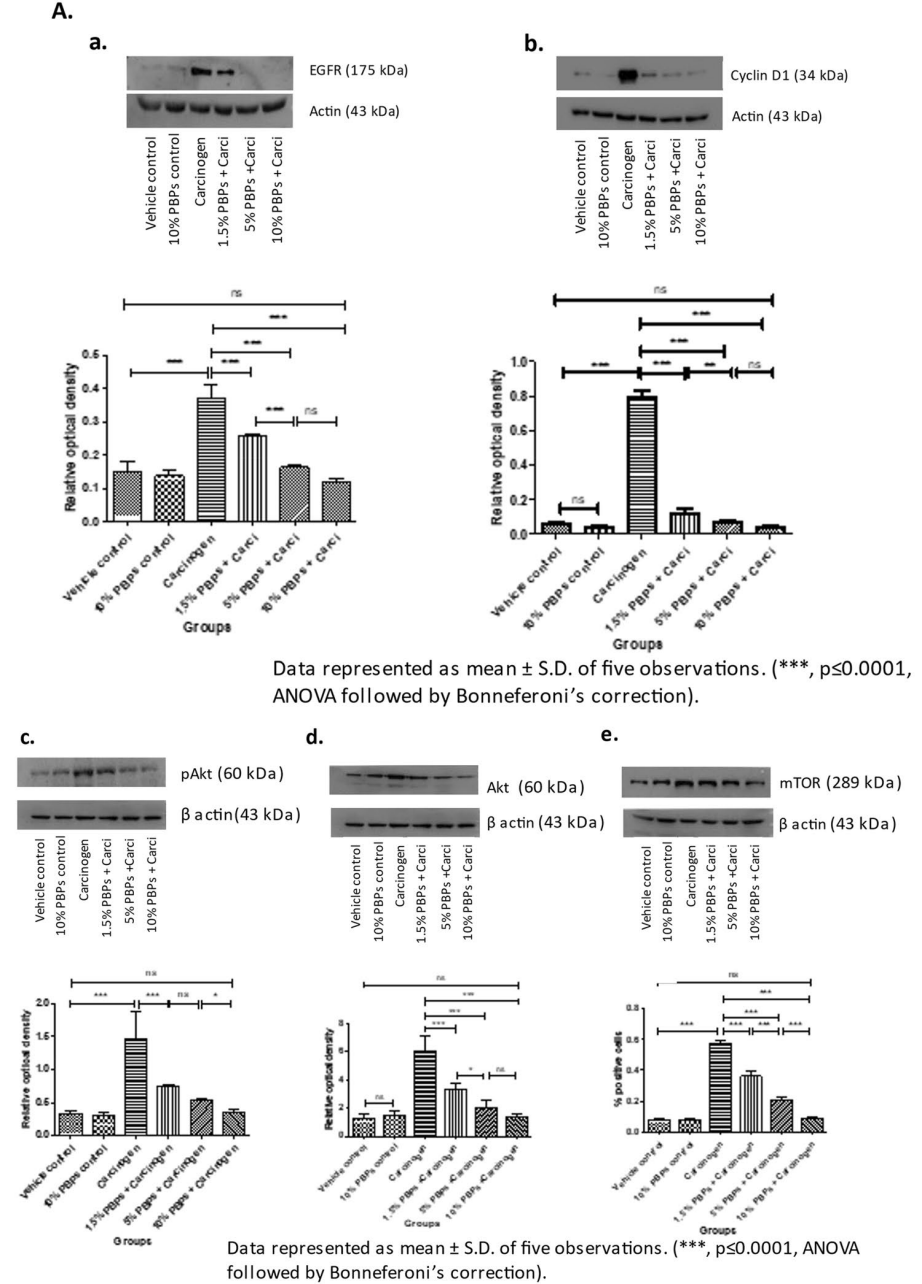

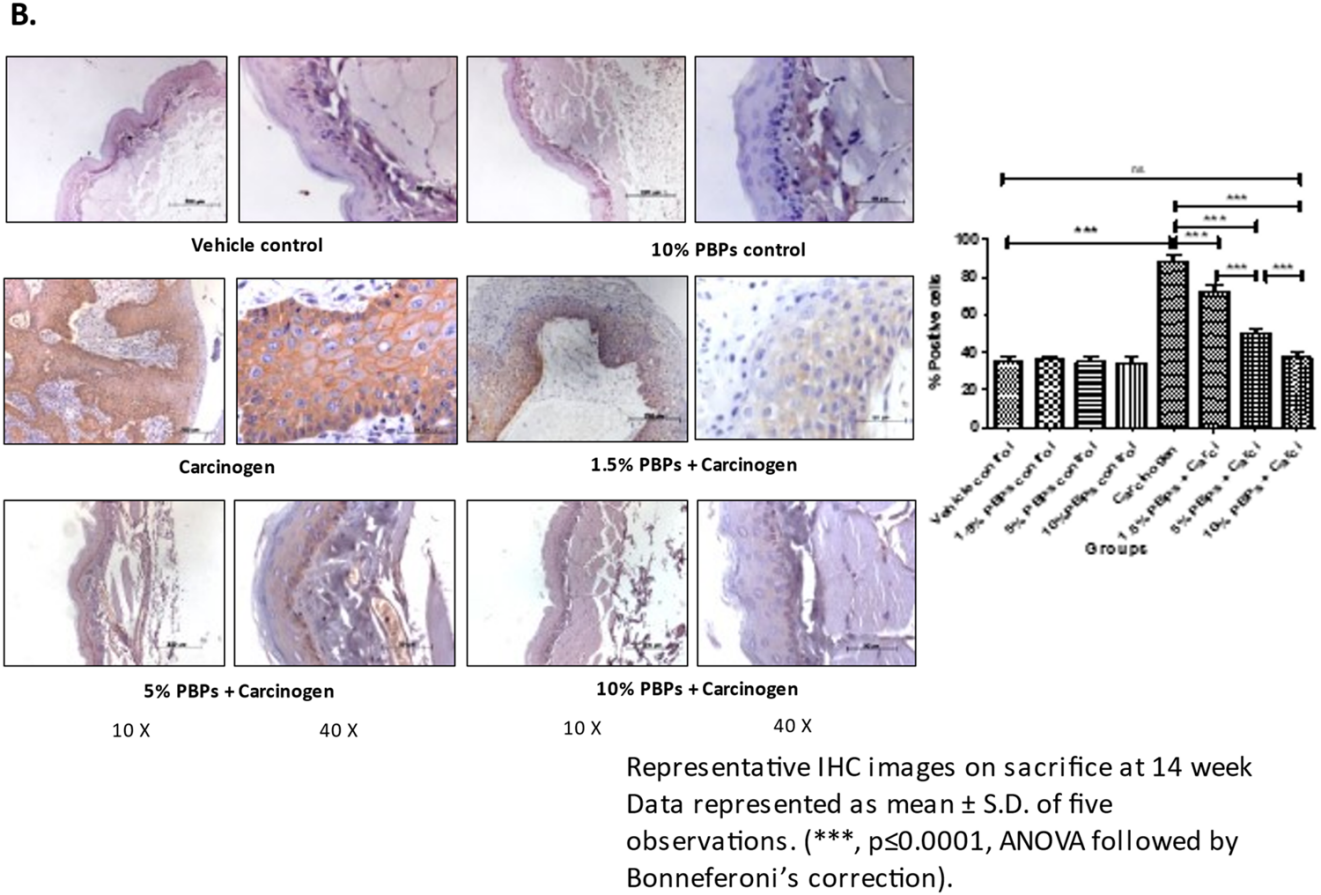
Effect of pre and concurrent treatment of different doses of PBPs on EGFR and its downstream targets in experimental oral cancer. (**A**) Representative blots and relative densitometric levels of (**a**) EGFR, (**b**) Cyclin D1, (**c**) Akt, (**d**) pAkt and (**e**) mTOR. Data represented as mean ± S.D. of five observations. (***, *p* ≤ 0.0001, ANOVA followed by Bonneferoni’s correction). (**B**) Representative photomicrographs showing immunohistochemical detection of EGFR. Data represented as mean ± S.D. of five observations. (***, *p* ≤ 0.0001, ANOVA followed by Bonneferoni’s correction).

Expression of EGFR and Cyclin D1 was not significantly different in all controls (Supplementary Fig. 9B and C). In the carcinogen group expression of EGFR and Cyclin D1 was significantly increased as compared to its respective vehicle control group. PBPs treatment resulted in a decrease in expression of EGFR and Cyclin D1 in a dose-dependent manner. The expression of both EGFR and Cyclin D1 in the 10% PBPs + carcinogen group matched the expression of EGFR and cyclinD1 respectively in-vehicle control group (Fig. 3A a and b). The observed difference in expression of EGFR between vehicle control and carcinogen group; also dose-dependent decreased expression of EGFR in PBPs + carcinogen group was validated by IHC analysis (Fig. 3B).

#### PBPs decreased carcinogen-induced expression of signaling molecules- Akt and mTOR

Expression of pAkt, Akt, and mTOR in control groups was minimum and there was no statistically significant difference in them (Supplementary Fig. 9H and I). However, upon carcinogen treatment, the expression of pAkt, Akt, and mTOR increased significantly than their respective vehicle control groups. Exposure of PBPs decreased overexpression of pAkt, Akt, and mTOR in a dose-dependent manner significantly compared to their respective carcinogen-treated groups. Also, expression of pAkt, Akt, and mTOR in the 10% PBPs + carcinogen group and vehicle control were similar (Fig. 3A c–e).

### Pre and concurrent treatment of PBPs decreased carcinogen-induced expression of HIF-1α and VEGF

**Figure 4:**
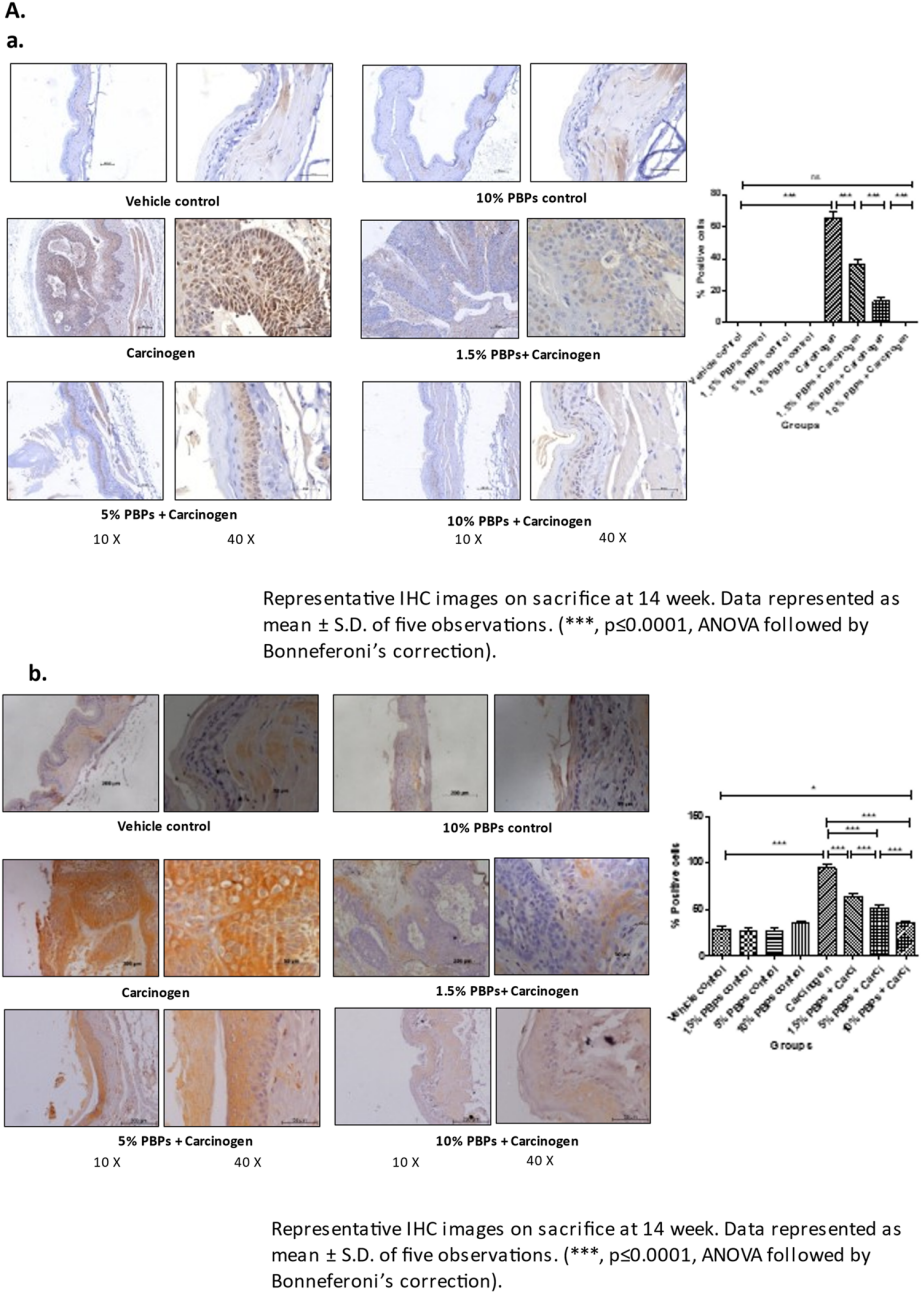

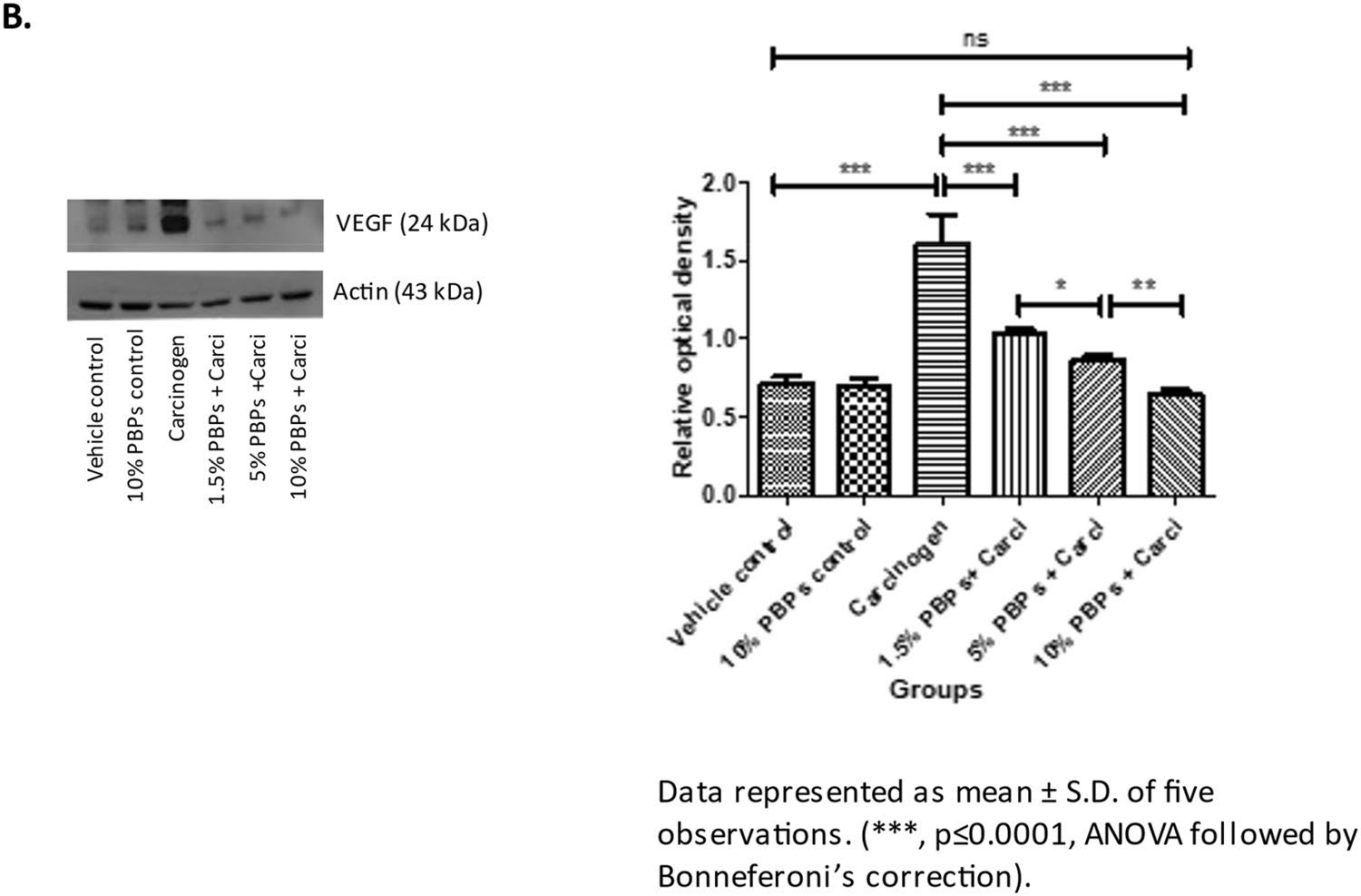
Effect of pre and concurrent treatment of different doses of PBPs on hypoxia and angiogenesis in experimental oral cancer. (**A**) Representative photomicrographs showing (**a**) HIF1-α and (**b**) VEGF staining. Data represented as mean ± S.D. of five observations. (***, *p* ≤ 0.0001, ANOVA followed by Bonneferoni’s correction). (**B**) Representative blots and relative levels of VEGF protein in buccal pouch total cell lysate. Data represented as mean ± S.D. of five observations. (***, *p* ≤ 0.0001, ANOVA followed by Bonneferoni’s correction).

IHC analysis in HBP tissue showed that expression of HIF1α and VEGF was minimum in control and the difference was statistically non-significant. In the carcinogen-treated group expression of HIF1α and VEGF increased significantly and was maximum. Increasing doses of PBPs decreased expression of HIF1α and VEGF in HBP. The difference in expression of HIF1α between vehicle control and 10% PBPs + carcinogen group was not significant. However, in-spite PBPs mediated decrease in expression of VEGF, its expression in 10% PBPs + carcinogen was significantly higher than that of the vehicle control group (Fig. 4A a and b). The difference in expression of VEGF protein across different treatment groups and its PBPs mediated dose-dependent downregulation was further confirmed by western blotting analysis (Supplementary Fig. 9G, Fig. 4B). Thus, the observed decreased expression of HIF1α and VEGF by PBPs suggests their anti-hypoxic and anti-angiogenic role.

### Correlation analysis between different molecular markers involved in the process of HBP oral carcinogenesis

Western blotting analysis of HBP tumor tissue sample from the carcinogen-treated group revealed a correlation between different molecular markers involved in various key cellular processes of oral carcinogenesis. Relative optical densities of a. Pro-apoptotic protein Bax and Pro-inflammatory protein COX-2 were found to be highly correlated (R = 0.9 and *p*-value = 0.04); b. Both the proliferation markers EGFR and PCNA were found to be highly correlated (R = 0.87 and *p*-value = 0.053); c. also strong correlation was observed between angiogenesis marker VEGF and downstream effector of EGFR molecule, pAkt (R = 0.9 and *p*-value = 0.04) (Supplementary Fig. 8). We have also evaluated correlation analysis among different biomarkers analyzed by both western blotting and IHC in 1.5% PBPs + carcinogen group, 5% PBPs + carcinogen group, and 10% PBPs + carcinogen group. We observed a direct correlation significant at the 0.01 level (2-tailed) in between all the biomarkers except Bax. While we observed inverse correlation significant at the 0.01 level (2-tailed) between bax and other biomarkers (Supplementary text 5).

## Discussion

In this study we have the first time demonstrated the chemopreventive efficacy of PBPs in experimental oral carcinogenesis. At all doses including the highest dose, we did not observe any PBP-mediated cytotoxicity as even after continuous exposure to highest dose of PBPs i.e. 10% PBPs for 14 weeks, at end of experiment the body weight of animals from 10% PBPs control group and 10% PBPs + carci group were increased as compared to their initial body weights similar to animals weights from vehicle control group with no statistically significant difference (Supplementary Fig. 2). Also, IHC and WB analysis revealed that there is no cytototoxicity as expression of proliferation, inflammation, oxidative DNA damage and apoptosis molecular markers remains unchanged between all different (1.5%, 5% and 10%) PBPs control group and vehicle control group with no statistically significant difference (Fig. 2B a–e) (Supplementary Fig. 9A, C, D, E, and F).

**Figure 5:**
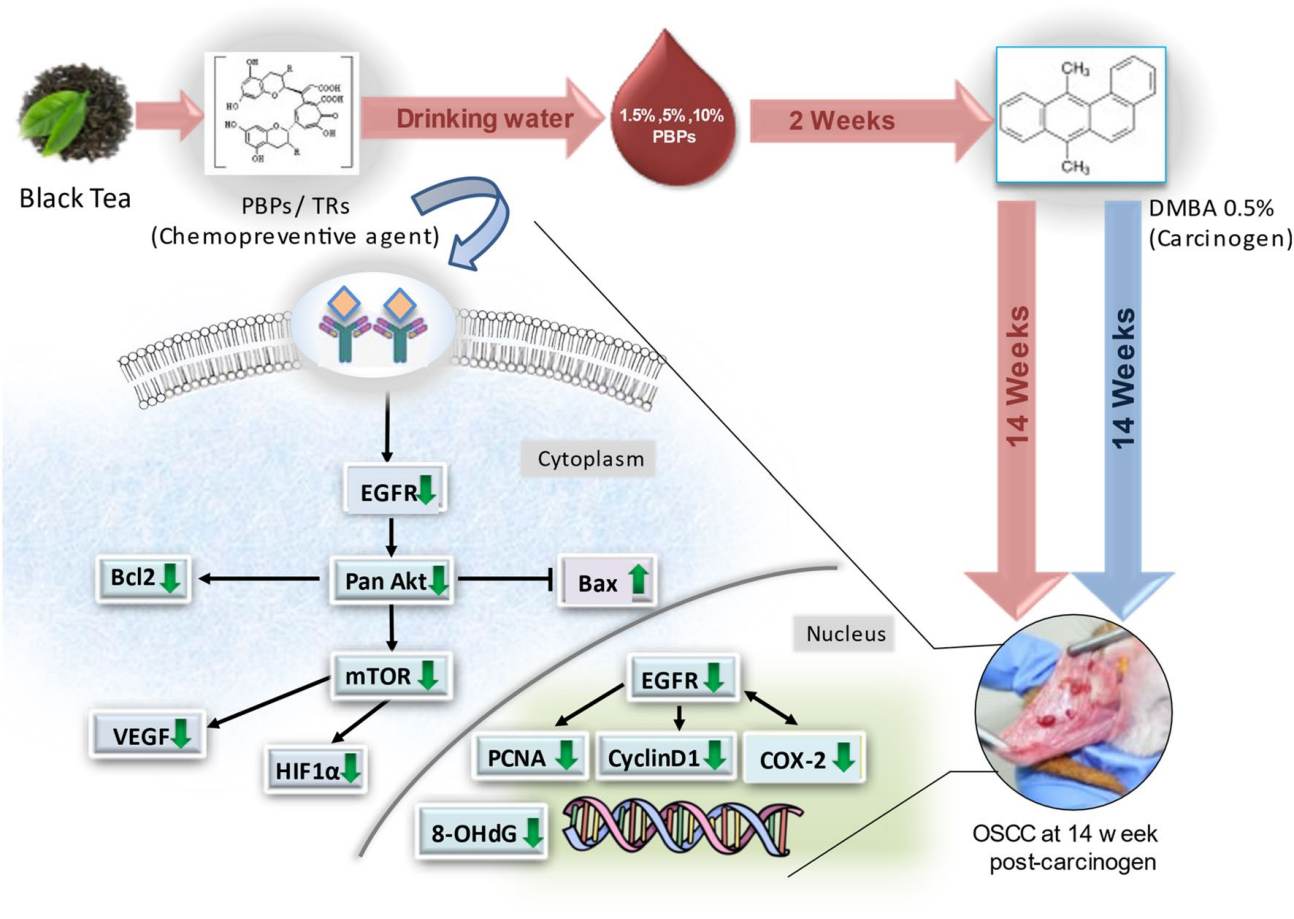
Schematic of the treatment plan and brief presentation of effect of PBPs on key biomarkers involved in process of oral carcinogenesis.

We have demonstrated that PBPs decrease macroscopic tumor number, volume, and burden in a dose-dependent manner. At the highest (10%) PBPs dose, we observed both no macroscopic and microscopic tumor development which demonstrates their strong chemopreventive property. Administration of PBPs decreased oxidative DNA damage, inflammation, proliferation, and increased apoptosis indicating its chemopreventive potential. For the first time in this report, the effect of PBPs exposure on EGFR and its major downstream target was evaluated. In our study, we have demonstrated by both IHC and western blotting overexpression of EGFR in tumor tissue and its dose-related downregulation by PBPs. PBPs have also decreased carcinogen-induced overexpression of EGFR downstream signaling targets- Akt and mTOR. Further, PBPs have decreased carcinogen-induced hypoxia and angiogenesis which together explains the observed underlying molecular basis of chemoprevention (Fig. 5).

Polyphenolic compounds in green tea or EGCG are extensively explored on oral cancer and their molecular targets are known^8–10^. However black tea which is the most consumed beverage after water^29^ has not been explored. Few studies have been conducted so far using polymeric black tea polyphenols (PBPs) despite being abundantly found in black tea. We have earlier demonstrated that PBPs decrease inflammation, proliferation, increase apoptosis and modulate the expression of Akt in experimental lung carcinogenesis^17,20^. In addition, PBPs have been reported to prevent colon carcinogenesis via modulation of the Wnt/beta-catenin pathway^19^. Also, PBPs have been reported to decrease proliferation and thereby prevented skin carcinogenesis^30^. All chemoprevention studies using black tea polyphenols in experimental oral carcinogenesis have used two commercial formulations—polyphenon B and BTF-35 to date. Polyphenon B and BTF-35 are mainly composed of monomeric polyphenols like EGCG, EGC; oligomeric polyphenols or theaflavins, and caffeine^31,32^. However, polyphenon B and BTF-35 do not contain major black tea ingredients—PBPs (47% of total polyphenols). Our results showing a decrease in proliferative index and increase in apoptosis are consistent with previous reports on polyphenon B in a hamster model of oral carcinogenesis^33^. To the best of our knowledge, this is the first report wherein chemopreventive efficacy of PBPs is evaluated in oral carcinogenesis and systematic dose-related effects are reported. Since the exposure to PBPs was the only variable in our study and as MALDI-TOF analyses of PBPs extract used in our study has demonstrated them to be free of other biologically active black tea-derived contaminants (e.g., caffeine, catechins, theaflavins) observed chemopreventive activity could be attributed to PBPs^17^. In the present study, administration of PBPs was 2 weeks before carcinogen treatment and during 14 weeks of carcinogen exposure, hence observed chemopreventive effects can be due to both anti-initiation as well as anti-promotion activity of PBPs. Due to both pre and concurrent exposure of PBPs, it is not possible to attribute the observed biological activity to only parent PBPs or metabolic products derived from PBPs, and hence it can be due to one and/or both of them. Diet employed in our study is majorly plant-derived (composition of the diet is given in Supplementary text 2. I). Plant-derived diets are known to delay the spontaneous or carcinogen-induced tumors because they induce the production of Phase II metabolizing enzymes which are responsible for conjugation reaction and excretion of carcinogenic metabolites^34,35^. Significant chemopreventive activity attributable to PBPs was demonstrated despite using a plant-derived diet; which suggests that the chemopreventive effect of PBPs would have been pronounced if a synthetic diet devoid of any chemopreventive agents was used.

EGFR expression is related to disease progression from nonmalignant leukoplakia to invasive disease^36,37^. EGFR is an important druggable target in HNSCC and its activation is associated with the malignant phenotype, inhibition of apoptosis, and increased metastatic potential^21,38^. This explains the need of finding novel, safe chemopreventive agents which can modulate the EGFR pathway. It is known that green tea polyphenol EGCG downregulates the overexpression of EGFR in oral cancer cell lines^39^. Furthermore, EGCG in combination with Erlotinib, a small molecular inhibitor of EGFR strongly induces cell cycle arrest in HNSCC cell lines^40^. Apart from tea, another chemopreventive agent Apigenin has been reported to decrease expression of EGFR, ErbB2, Erk1/2, and Akt in HNSCC cell lines^41^. There is only one study available to date wherein EGFR and pEGFR (Tyr1173) expression was reported to be decreased by a mixture of Chinese herbs- antitumor B in a mice model of oral carcinogenesis however, further downstream signaling kinases were not evaluated^42^. Detailed EGFR pathway analysis and systematic dose–response study using PBPs/ TRs as a chemopreventive agent in animal model systems are lacking. Ours is the first study to demonstrate dose-dependent PBPs mediated decrease in carcinogen-induced overexpression of EGFR and its downstream signaling kinase- Akt and mTOR. Also, many preclinical studies have shown that most inhibitors of EGFR also work by inhibiting HIFα and its related molecules^43,44^. The observed downregulation of EGFR and CycllinD1 by PBPs reported in our study is thus interlinked to downregulation of HIF1α and VEGF by PBPs. Our lab has demonstrated predictive and prognostic values of HIF1 alpha in HNSCC^45^. This is the first report wherein we showed downregulation of EGFR and interrelated biomarkers—CyclinD1, pAkt, Akt, mTOR, HIF1α, and VEGF in a dose-dependent manner with PBPs exposure is studied. The observed differences in the expression of signaling kinases suggest the chemopreventive potential of PBPs via modulation of the EGFR pathway.

Various plant or plant-derived products or purified phytochemicals have shown their chemopreventive efficacy in experimental oral carcinogenesis via modulation of a variety of transcription factors, many xenobiotics metabolizing enzymes, etc. Most of them show pleiotropic effects and are less toxic in their original form. The majority of these studies have utilized DMBA induced HBP carcinogenesis model to test the effect of various chemopreventive agents with varying doses, duration, and routes^5^. The reason for the popularity of the model system lies in the similarity with the human sequential progression of disease from pre-invasive lesions to invasive OSCC; similar histopathological, genetic, epigenetic changes/alterations as seen in human OSCC. However, the HBP model also has certain limitations such as HBP is an immunologically privileged site; human buccal mucosa is much thicker than HBP; tumors developed in hamsters are exophytic while human oral tumors are majorly endophytic^5^. Limited information is available globally on the structure and characterization of PBPs. Currently available techniques are unable to detect PBPs-related signals in tissue and serum samples. Hence bioavailability and pharmacokinetic studies using PBPs are not possible which can be considered as a limitation. Serum-based biomarker analysis can offer complementary information on the development of robust predictive biomarkers in the process of oral carcinogenesis. We first time tried to find a correlation between different molecular markers based on western blotting tissue molecular marker expression which can be used as predictive markers in process of oral carcinogenesis and chemoprevention. However, their utility as predictive biomarkers needs to be confirmed by conducting preclinical studies in the future wherein molecular marker expression will be assessed in both tissue and serum samples. As in the current study, PBPs have shown promising chemopreventive potential in pre and concurrent treatment settings, a study involving evaluation of the chemopreventive potential of PBPs in post-treatment settings is warranted.

In summary, our study illustrates promising chemopreventive biological effects of major PBPs/TRs in experimental oral carcinogenesis to which a large number of people are exposed regularly. PBPs show a protective effect against oral carcinogenesis possibly via modulating EGFR and its downstream pathway. Abuse of tobacco is an important risk factor for oral cancer. However, primary prevention strategies involving limiting exposure to tobacco have minimal success due to the addictive nature of nicotine and economic, individual freedom-related issue. Little change is observed in the prevalence of chewing tobacco use between 1990 and 2019. In the absence of primary prevention, tobacco habits will increase over the coming decades^1,2^. Thus, efforts should be in direction of a cost-effective chemopreventive agent identification because interventions based on plants or plant products or purified phytochemicals have advantages in terms of their ease of production and long known history of exposure.

## Supplementary Information

Supplementary Information.

## Data Availability

All datasets on which the conclusions of the paper rely is presented in the main manuscript or additional supporting files whenever possible and available to readers. We assure that after publication in the journal, materials described in the manuscript, including all relevant raw data, will be freely available to any researcher wishing to use them for non-commercial purposes, without breaching participant confidentiality.

## Abbreviations

HNSCC: Head and neck squamous cell carcinoma
OSCC: Oral squamous cell carcinoma
HBP: Hamster buccal pouch
DMBA: 7,12-Dimethylbenz[a]anthracene
EGFR: Epidermal growth factor receptor
PBPs: Polymeric black tea polyphenols
TF: Theaflavins
EGCG: Epigallocatechin gallate
8-OHdG: 8-Hydroxy-2' -deoxyguanosine
PCNA: Proliferating cell nuclear antigen
COX-2: Cyclooxygenase-2
Bcl-2: B-cell lymphoma 2
HIF1- alpha: Hypoxia-inducible factor 1-alpha
VEGF: Vascular endothelial growth factor
Akt: Protein kinase B
pAkt: Phospho-protein kinase B
mTOR: Mammalian target of rapamycin
HRP: Horseradish peroxidase
DAB: 3,3′-Diaminobenzidine
WB: Western blotting
IHC: Immunohistochemistry
TLC: Thin-layer chromatography
MALDI-TOF: Matrix-assisted laser desorption/ionization-time of flight
