# Supplementary material for: Prevention of carcinogen-induced oral cancers by polymeric black tea polyphenols via modulation of EGFR-Akt-mTOR pathway
Source: Sci Rep. 2022 Aug 25;12:14516. doi: 10.1038/s41598-022-18680-0 (PMC9411124; doi:10.1038/s41598-022-18680-0)

Prevention of carcinogen-induced oral cancers by Polymeric black tea polyphenols via modulation of EGFR-Akt-mTOR pathway

Vaishnavi K. Nimbalkar[1, 2], Jeet Gangar[1], Saptarsi Shai[1], Pallavi Rane[2,3], Subham Kumar Mohanta[2,3], Sadhana Kannan[2,3], Arvind Ingle[2,4], Neha Mittal[2,5], Swapnil Rane[2,6], and Manoj B. Mahimkar[1, 2*]

[1]Mahimkar Lab, Cancer Research Institute (CRI), Advanced Centre for Treatment, Research and Education in Cancer (ACTREC), Tata Memorial Centre (TMC), Kharghar, Navi Mumbai, India

[2]Homi Bhabha National Institute, Training school complex, Anushakti Nagar, Mumbai-400085, India.

[3]Biostatistician, Clinical Research Secretariat, Advanced Centre for Treatment, Research and Education in Cancer, Tata Memorial Centre, Navi Mumbai, Maharashtra, India

[4]Laboratory Animal Facility, Advanced Centre for Treatment, Research and Education in Cancer (ACTREC), Tata Memorial Centre (TMC), Kharghar, Navi Mumbai, India

[5]Department of Pathology, Tata Memorial Hospital, Tata Memorial Centre (TMC), Parel, Mumbai, India

[6] Advanced Centre for Treatment, Research and Education in Cancer (ACTREC), Tata Memorial Centre (TMC), Kharghar, Navi Mumbai, India

*__Correspondence to__ Dr. Manoj B Mahimkar.

Associate Professor HBNI & Principal Investigator ACTREC

KS 106, Cancer Research Institute,

Advanced Centre for Treatment, Research and Education in Cancer,

Tata Memorial Centre, Kharghar,

Navi Mumbai - 410 210

INDIA

Email Address- mmahimkar@actrec.gov.in , manojbmahimkar@gmail.com .

Phone: 91-22-2740 5049 / 3043 5049

Mobile: 91-98206 65644

Fax: 91-22-2740 5085 / 5058

Supplementary text

1. Isolation and characterization of Polymeric Black tea polyphenols (PBPs)

I. Protocol for isolation of PBPs

PBPs extraction was carried out as per published protocol[1, 2]. Briefly, 450 g of black tea powder was decaffeinated with four litres of chloroform for eight days. The remaining tea powder in the thimble was extracted with four litres of ethyl acetate for eight days. Residual black tea powder a in thimble containing PBP-2,3,4 and 5 fractions were dried and stored at -20^{0}C. The ethyl acetate extract containing PBP-1, catechins, and theaflavins was concentrated using a flash evaporator (Borosil Glass Works Ltd., Mumbai, India). The concentrated extract was reconstituted with acetone (100 ml) and diethyl ether (800 ml) to precipitate PBP-1. The precipitate was further centrifuged, dried, and stored at -20^{0}C. The batch-wise details of PBPs extraction are given in (supplementary table 1).

Supplementary Table 1 (ST1): Polymeric black tea polyphenol extraction batchwise yield of all five fractions- 1,2,3,4 and 5.

Initial Weight of Tea Powder (g)	Weight of Tea Powder after decaffeination (g) mean±SD	Caffeine Content (g)	Residual black tea powder containing PBP 2,3,4,5 fractions (g)	Weight of PBP 1 fraction (g)
450	438.67±3.75	11. 41 ±3.90	417.50+6.61	3.32+2.09

Batch No	Initial weight of tea powder (g)	Weight of tea powder after caffeine extraction (g)	Caffeine content (g)	Weight of residual tea powder containing PBP fractions-2,3,4,5 (g)	Weight of PBPs fraction1 (g)
1	450.00	440.00	10.00	403.30	2.32
2	450.00	442.00	8.00	429.00	4.60
3	450.00	442.00	8.00	421.10	7.40
4	450.00	445.00	5.00	426.70	2.70
5	450.00	438.00	12.00	419.00	4.42
6	450.00	441.50	8.50	416.50	5.75
7	450.00	440.00	10.00	426.50	1.00
8	450.00	441.00	9.00	418.50	3.00
9	450.00	440.00	10.00	422.5	1.00
10	450.00	440.70	9.30	417.10	3.00
11	450.00	440.00	10.00	421.5	5.00
12	450.00	441.00	09.00	407.00	2.00
13	450.00	434.00	16.00	410.50	1.00
14	450.00	437.0	13.00	414.50	1.00
15	450.00	433.50	16.50	416.20	2.00
16	450.00	433.30	17.00	415.00	1.63
17	450.00	431.00	19.99	415.00	7.00
18	450.00	436.00	14.00	416.00	5.00

II. Characterization of PBPs extract by TLC and MALDI-TOF

A. Protocol for MALDI-TOF

- MALDI_TOF analysis of PBPs extract was carried out as per published protocol[1].
- Samples were analyzed using a-cyano-4-hydroxycinnamic acid (HCCA) matrix.
- Samples- PBPs, caffeine, EGCG standard, and Theaflavin standard were dissolved in 0.1% trifluoroacetic acid (TFA) in 50% acetonitrile (ACN) solvent at a concentration of 1mg/20µl. PBPs were mixed with HCCA matrix in a 1:1 ratio and this mixture was loaded (2µl) on a 396array plate which was further air-dried at room temperature.
- MALDI-TOF mass spectra were acquired using Bruker Daltonics Ultra flex-II, on a reflectron mode. The resulting MS data were analyzed using Flex analysis 3.0 (Brucker Daltonic, Bremen, Germany) software.

B. Instrumentation and calibration details of MALDI TOF

Compounds co-crystallized with the matrix are desorbed and ionized by a solid-state laser Neodymium/Yettrium aluminium garnet [Nd-YAG] (wavelength 337 nm; 4 ns pulse width) and extracted by 4 kV pulse voltage with time-delayed extraction of 70 ns before entering the time-of-flight mass spectrometer and accelerated under 20 kV. All spectra are recorded with a detector voltage of 2.5 kV and are the average result of 800 laser shots. The laser intensity and sensitivity of the detector are variable. MALDI-TOF is calibrated with HCCA matrix as a two-point internal calibration [M+H]2+ m/z 190.04987 and [2M+H] + m/z 37.09246. In the interpretation of spectra, peaks at m/z range 100 to 2500 are taken into account.

C. Evaluation of purity of PBPs extract

To ascertain that each extracted batch of PBPs is free from other components of black tea i.e., caffeine, EGCG (monomeric black tea polyphenols), and theaflavins (oligomeric black tea polyphenols), they were run together on a silica plate using thin-layer chromatography (TLC). PBPs remain bound to the matrix at the origin where they were loaded while all other components migrate with the running solvent system and get resolved. The absence of any mobile compound in PBPs shows that it is free from these contaminants. Further, this TLC-based analysis was confirmed by using MALDI- TOF technique. Commercially available compounds such as caffeine (Sigma C0750), EGCG (Sigma E4143), and theaflavins (SigmaT5550) were used in both TLC and MALDI-TOF experiments as standards. MALDI-TOF spectra with a-cyano-4-hydroxycinnamic acid (HCCA) as a matrix, shows EGCG (m/z 459.11), caffeine (m/z 195.03), and theaflavin (m/z 565.40) specific peaks which match with those reported (Supplementary figure 1). PBPs extract shows the presence of none of these contaminants whereas each PBPs sample showed two PBPs specific peaks of (m/z 855.41) and (m/z 877.43) similar to a previously published report from our lab (Supplementary figure 1) [1]. Extracted PBPs are free from known biologically active components like caffeine, EGCG, theaflavin, and thus observed biological activity is attributed to only PBPs.

III. Preparation of different doses of PBPs

Each extracted batch of PBPs was characterized by TLC and MALDI-TOF and used for the preparation of different doses. For use, residual black tea powder containing PBP-2,3,4,5 and PBP-1 was boiled in autoclaved MiliQ to prepare 10%, 5%, 3% and 1.5% PBP extract doses in definite proportions. After preparing doses each time, total solids (mg/ml) in every dose of

PBPs were measured to confirm their dose-related yield. The details are given in the following table-

Supplementary Table 2 (ST2): Preparation of different doses of PBPs and their dry weight analysis

PBPs dose concentration (%)	Weight of residual black tea powder containing PBPs-2,3,4 and 5 (g)	Weight of PBP-1 (g)	Final Volume of PBPs dose (ml)	PBPs dry weight (mean ± SD) (mg/ml)
10%	64.86	2.00	1000	38.60±5.50
5%	32.43	1.00	1000	17.70±3.75
3%	19.46	0.60	1000	12.13±1.42
1.5%	9.73	0.30	1000	6.96±2.68

2. Details of animal study

I. Diet composition

The diet used to feed hamsters throughout the study period was Altromin 1324 P – Maintenance diet for rats/ mice - phytoestrogen deficient. The composition of the diet is- vegetable by-products, cereals, minerals, oils, and fats. Diet contains crude nutrients as follows-

Supplementary Table 3 (ST3): Composition of animal diet

Diet components	% Composition
Crude protein	19.20
Crude fat	4.10
Crude fiber	6.10
Crude ash	6.90

The diet was stored in dark and dry conditions. The animals were fed libitum with the diet.

II. Animal treatment plan

- Male hamsters, 6-8 weeks old received from Laboratory Animal Facility, ACTREC were randomized into ten groups.
- PBPs control (1.5% PC, 3% PC, 5% PC and 10% PC) and PBPs+carcinogen (1.5% P+C, 3%P+C, 5% P+C and 10% P+C) groups were primed with black tea derived PBPs rich extract (1.5%, 3%, 5% and 10%) as only source of drinking water for two weeks. While vehicle control (VC) and Carcinogen (C) group animals received plain drinking water for two weeks ad libitum.
- Right buccal pouch of all animals in vehicle control (VC) and PBPs control (1.5% PC, 3% PC, 5% PC, and 10% PC) group were topically applied with glyceryl trioctanoate and continued receiving plain drinking water and black tea derived PBPs rich extract (1.5%, 3%, 5%, and 10%) respectively for next fourteen weeks.
- Right buccal pouch of animals in carcinogen (C) and PBPs+carcinogen (1.5% P+C, 3%P+C, 5% P+C, and 10% P+C) groups were topically applied with 0.5% DMBA dissolved in glyceryl trioctanoate and continued on plain drinking water and PBPs (1.5%, 3%, 5%, 10%) respectively for fourteen weeks (Figure 1).
- Throughout the experiment, paintbrush no. 4 was used to apply DMBA and glyceryl trioctanoate on the right buccal pouch of the hamster, which applies ≈ 60 µl quantity. Various PBPs doses and plain drinking water was administered ad libitum.

- Body weights of all animals were monitored every week till sacrifice Supplementary figure 2.
- All animals were euthanized in a CO_2 chamber after fourteen weeks of DMBA treatment.

III. General observation

Throughout the experiment, hamsters from different treatment groups drank an equal quantity of water and different doses of PBPs- 1.5%, 3%, 5%, and 10%. The details are given in following table

Supplementary Table 4 (ST4): Consumption data of water and PBPs by hamster

Dose (%)	Consumption (mean±SD) (ml)
Water	7.12±6.5
1.5%	7.30±5.9
3.0%	7.62±7.5
5.0%	7.52±9.5
10.0%	7.37±7.4

*Mean $\pm$ SD is based on 16-week water consumption data

In the entire study, no signs of toxicity were observed in any treatment group and there was no mortality in the treatment group. Images of the gross appearance of tumors after 14 weeks of carcinogen treatment are provided in (Supplementary figure 2). Images of H&E stained HBP tissue sections showing the sequential progression of disease are provided in (Supplementary figure 3). Final body weights were higher than initial body weights with no statistical significance indifference across the groups (Table 1 and Supplementary figure 2).

IV. Macroscopic tumor volume and tumor burden calculation

Tumor volume and burden were calculated as per established protocol[3]. Tumor volume (V) was calculated using formula $V= 4/3 \pi r^3$, wherein r is the radius of tumor which is calculated by the formula $(d_1/2+d_2/2)/2 = r$, where $d_1$and d_2 are minimum and maximum diameters of tumors. Diameters of tumors were measured by using a digital vernier caliper. Tumor burden was calculated as average tumor multiplicity * average tumor volume.

V. Microscopic tumor number and tumor area calculation

- Briefly, 10% buffered formalin-fixed entire buccal pouch tissues were processed through a series of graded alcohol and finally embedded in a paraffin block.
- Each tissue block was subjected to 5µm thickness tissue sections. Every section at 50µm interval was stained with haematoxylin and eosin (H&E).
- Each stained section was tile scanned at a total 100X magnification with LSM 510 Meta Carl Zeiss confocal microscope (Zen software 2012, SP1, Zeiss, Jena, Germany).
- Tile scans were graded by two independent pathologists who were blinded for treatment groups into different lesions as hyperplasia, dysplasia, and squamous cell carcinoma[4].
- The total number of each type of lesion was counted for all tile scans of a single hamster. An average number of lesions for each lesion type was calculated for three animals.
- The area of each proliferative lesion was marked by two independent pathologists on each tile scan. These tile scans were evaluated by Image J software (Rasband, W.S., Image J, U.S. National Institutes of Health, Bethesda, MD, http://imagej.nih.gov/ij/, 1997–2015).
- Image J was pre-calibrated for conversion of pixels into square micrometer (μm^2) according to magnification used for image acquisition (100X).

- Each lesion was outlined by a hand-free tool and its area was measured as μm^2. Area measurement of each type of lesion of all tile scans for an individual hamster was carried out. The average area for each type of lesion for three hamsters was calculated.

VI. **Supplementary Table 5 (ST5):** Comparison between microscopic tumor count and macroscopic tumor count

Group	Animal No.	Microscopic tumor			Macroscopic tumor Number
		Range	Median	Number	
Carcinogen	O92235	3 -7	4	4.35	7
Carcinogen	O92306	1 -5	5	4.11	7
Carcinogen	O92286	1 -7	4	4.45	6
1.5% P+C	O9/2245	1 -4	2	2.04	3
1.5% P+C	O9/2243	0 -3	2.5	2.08	3
1.5% P+C	O9/2244	0 -3	3	2.17	3
5% P+C	O9/2368	0 -2	1	1.00	2
5% P+C	O9/2370	1 -2	2	1.92	2
5% P+C	O9/2372	0 -2	2	1.18	2
10% P+C	O92437	0 -0	0	0.00	0
10% P+C	O92438	0 -0	0	0.00	0
10% P+C	O92440	0 -0	0	0.00	0

VII. **Supplementary Table 6 (ST6):** Comparison between microscopic tumor area and macroscopic tumor volume

Group	Animal No.	Microscopic SCC area	Macroscopic SCC volume
Carcinogen	O92235	1333609.59	9.66
Carcinogen	O92306	1200866.14	4.83
Carcinogen	O92286	1588133.94	7.24
1.5% P+C	O9/2245	477583.75	0.83
1.5% P+C	O9/2243	426606.17	2.15
1.5% P+C	O9/2244	436606.17	0.74
5% P+C	O9/2368	251314.96	1.93
5% P+C	O9/2370	286384.05	1.59

5% P+C	O9/2372	241708.24	1.56
10% P+C	O92437	0.00	0.00
10% P+C	O92438	0.00	0.00
10% P+C	O92440	0.00	0.00

3. Protocol for western blotting of whole cell lysate of HBP tissue

- Briefly, 50µg of total cell lysate protein was loaded and resolved on 8-12% SDS PAGE and transferred on polyvinylidene difluoride (PVDF) membrane (GE Amersham Biosciences, Buckinghamshire, UK). The blots were cut prior to hybridisation with the antibodies.
- The membrane was then blocked with 8% non-fat skimmed milk or 5% Bovine Serum Albumin (BSA) in Tris-buffered saline (TBS, pH 7.4) containing 0.1% tween 20 (PBST).
- The membrane was probed with primary antibodies of β-actin, PCNA, Cyclin D1, EGFR, Bax, Bcl2, VEGF, pAkt, Akt, and mTOR at their respective standardized dilutions for overnight at 4^0C (supplementary table 2).
- The membrane was washed with PBST for 10 minutes on a rocker. The membrane was then incubated with anti-mouse or anti-rabbit HRP labeled secondary antibodies at 1:2000 dilutions for 1 hour at room temperature.
- The membrane was then washed with PBST for 20 minutes on a rocker. Immunoreactive bands were visualized using a chemiluminescence reagent (Clarity Western ECL substrate, Western Bright ECL kit).

Supplementary Table 7 (ST7): Details of western blotting conditions

Marker	Source, Catalog No., Lot No.	Host species, Clone	Band position	Amount of protein loaded	Blocking	1^0Ab Conc.	TBST washes after 1^0	2^0Ab Conc.	TBST washes after 2^0
**Actin**	Santa Cruz, SC-1616, F2111	Rabbit, polyclonal	43kDa	30-50 µg	8% Milk	1:2000	10 min X 2	1:2000	10 min X 3
**PCNA**	Abcam, ab18197, GR3192341-1	Rabbit, polyclonal	29kDa (25-35kDa)	30µg	8% Milk	1:500	10 min X 2	1:2000	10 min X 3
**EGFR**	Cell signaling, 4267S, 19	Rabbit, monoclonal (D38B1)	175kDa	50µg	8% Milk	1:500	10 min X 2	1:2000	10 min X 3
**CyclinD1**	Abcam, ab13417, GR3212345-3	Rabbit, monoclonal (EPR2241)	34kDa	30µg	8% Milk	1:1000	10 min X 2	1:2000	10 min X 3

**Cox2**	Abcam, 15191, GR320409-6	Rabbit, polyclonal,	74kDa	30μg	5% Milk	1:1000	10 min X 2	1:2000	10 min X 3
**Bax**	Abcam, ab7977, GR98755-2	Rabbit, polyclonal	26kDa (25-35kDa)	30μg	8% Milk	1:500	10 min X 2	1:2000	10 min X 3
**Bcl2**	Abcam, ab7973, GR99542-3	Rabbit, polyclonal	25kDa	30μg	8% Milk	1:500	10 min X 2	1:2000	10 min X 3
**VEGF**	Abcam, ab1316, GR41450-46	Mouse, monoclonal (VG-1)	23kDa	50μg	8% Milk	1:500	10 min X 2	1:2000	10 min X 3
**Akt**	Cell signaling, 4691,17	Rabbit, monoclonal (C67E7),	60kDa	50μg	8% Milk	1:1000	10 min X 2	1:2000	10 min X 3
**pAkt (ser473)**	Cell Signaling, 4060T, 25	Rabbit, monoclonal (D9E),	60kDa	50μg	8% BSA	1:1000	10 min X 2	1:2000	10 min X 3
**mTOR**	Cell Signaling, 2983, 19	Rabbit, monoclonal (7C10)	289kDa	50μg	5% BSA	1:500	10 min X 2	1:2000	10 min X 3

4. Protocol for IHC staining of HBP tissue sections

- Tissue sections were deparaffinized by heating at 60^0C for 20 minutes followed by washing in xylene for 15 minutes at room temperature.
- The sections were then rehydrated by passing through graded series of alcohol and finally rinsed in distilled water.
- Antigen retrieval conditions for each molecular marker were standardized using different combinations of antigen retrieval buffers with heating conditions, serum blocking with different concentrations, primary antibody dilutions, and washing conditions (supplementary table 3).
- Antigen retrieval buffers used were Tris-EDTA buffer (10 mM Tris base, 1 mM EDTA, 0.05% Tween 20, pH 9.0) or Sodium citrate buffer (10 mM Sodium citrate, 0.05% Tween 20, pH 6.0) or EDTA buffer (1 mM EDTA, 0.05% Tween 20. pH 8.0).
- Endogenous peroxidase activity in tissue sections was quenched by incubating them in 3% H_2O_2 in methanol for 1 hr in dark at room temperature.
- Blocking with horse serum at different concentrations was done at room temperature as per standardization in a humidified chamber to avoid non-specific binding by primary antibodies.
- Tissue sections were then incubated with different dilutions of primary antibodies for 8-OHdG, PCNA, COX-2, EGFR, VEGF, Bax, Bcl2, and HIF1α overnight at 4^0C in a humidified chamber.
- The primary antibody was replaced with an IgG isotype antibody for negative control or isotype control. Both sections having primary antibody and IgG isotype control were processed simultaneously.
- Tissue sections were then washed with PBST (PBS, pH 7.4, 0.05%- 0.1% Tween 20) as per standardization on a rocker.
- Tissue sections were incubated with 1: 100 diluted anti-rabbit or anti-mouse biotinylated secondary antibodies for 1 hour at room temperature in a humidified chamber.
- Detection was conducted using a Vectastain ABC system kit (Vector Laboratories, Burlingame, CA). Diaminobenzidine (DAB) was used as chromogenic substrate and Mayor's haematoxylin was used as counterstain.
- The slides having stained tissues were dehydrated using graded alcohol series and xylene. The tissues were then mounted using DPX and slides were allowed to dry.
- Images were captured using Zeiss Microscope (Imager Z1) which was attached to an Axiocam MRc5 digital camera.
- For IHC of VEGF protein in fixed tissue sections of hamster buccal pouch, Dako envision high pH antigen retrieval kit was used. With every batch of IHC, a positive control tissue was included.

Supplementary Table 8 (ST8): Details of IHC conditions

**Marker**	**Source and Catalogue No**	**Host species, clone**	**Antigen Retrieval Buffer**	**Antigen retrieval**	**Blocking**	**1°Ab Conc.**	**PBST washes**	**Positive control**
**8OHdG**	Abcam, ab48508, GR3408439-1	Mouse, monoclonal	Tris EDTA pH 9 + 250µl Tween 20	700 powers; (6 min oven + 10 min A.C.) X2	7% Horse serum 2 hrs	1:50	1 lit PBS+ 1ml Tween 20; x 3 (10 min)	Human colon

**PCNA**	Abcam, ab18197, GR3192341-1	Rabbit, polyclonal	Tris EDTA pH 9 + 250µl Tween 20	700 powers; (2min oven + 2 min A.C.) X2	3% Horse serum 1 hr	1:450	1 lit PBS+ 1ml Tween 20; x 3 (10 min)	Rat Brain
**COX2**	Abcam, ab15191, GR320409-6	Rabbit, polyclonal	Sodium Citrate + 250 µl Tween 20	700 powers; (6 min oven + 6 min A.C.) X2	3% Horse serum 1 hr	1:50	1 lit PBS+ 0.5ml Tween 20; x 3 (10 min)	Human Breast cancer
**EGFR**	Cell signalling, 4267S, 19	Rabbit, monoclonal (D38B1)	Tris EDTA pH 9 + 250µl Tween 20	700 powers; (6 min oven + 10 min A.C.) X2	3% Horse serum 1 hr	1:50	1 lit PBS+ 0.5 ml Tween 20; x 2 (10 min)	Human HNSCC
**VEGF**	Abcam, ab1316, GR41450-46	Mouse, monoclonal (VG-1)	Sodium Citrate + 250 µl Tween 20	90^0 C in prewarm buffer in water bath	Dako	1:50	Dako	Mouse kidney
**Bax**	Abcam, ab7977, GR98755-2	Rabbit, polyclonal	Tris EDTA pH 9 + 250µl Tween 20	(560 power- 5 min, 700 power-10 min, cool-10 min) X1	5% Horse serum 1 hr	1:50	1 lit PBS+ 0.5 ml Tween 20; x 2 (10 min)	Human LI cancer CK9453
**BCl2**	Abcam, Ab7973, GR99542-3	Rabbit, polyclonal	EDTA pH 8 + 250µl Tween 20	560 power- 5 min, 700 power-10 min, cool-10 min	5% Horse serum 1 hr	1:75	1 lit PBS+ 1ml Tween 20; x 2 (10 min)	Human lymph node
**HIF1 alpha**	Novus Biologicals, NB100479, AR	Rabbit, polyclonal	EDTA pH 8 + 250µl Tween 20	700 power- 6 min, 560 power-10 min, cool-10 min	15% Horse serum 2 hr	1:600	1 lit PBS+ 1ml Tween 20; x 3 (10 min)	Human Kidney tumor

5. Correlation analysis among 1.5% PBPs + carcinogen group, 5% PBPs + carcinogen group and 10% PBPs + carcinogen group for biomarker expression

A. Western blotting molecular marker analysis

Pearson_r_1.5_5_10_PBPs+Carcinogen										
	Cox2	PCNA	CyclinD1	EGFR	PanAkt	pAkt	mTOR	Bax	BCl2	VEGF
Cox2	1									
PCNA	.950**	1								
CyclinD1	.877**	.906**	1							
EGFR	.946**	.967**	.891**	1						
PanAkt	.903**	.852**	.824**	.907**	1					
pAkt	.942**	.987**	.890**	.957**	.887**	1				
mTOR	.968**	.967**	.858**	.965**	.915**	.971**	1			
Bax	-.973**	-.979**	-.877**	-.966**	-.895**	-.983**	-.969**	1		
BCl2	.964**	.891**	.821**	.874**	.888**	.900**	.926**	-.927**	1	
VEGF	.963**	.973**	.843**	.945**	.872**	.968**	.967**	-.977**	.934**	1
N = 15										
** Correlation is significant at the 0.01 level (2-tailed).										

B. IHC molecular marker analysis

Spearman_rho_1.5_5_10_PBPs+Carcinogen								
	Cox2	PCNA	OHdG8	Bax	BCl2	EGFR	H1F1	VEGF
Cox2	1							
PCNA	.943**	1						
OHdG8	.914**	.868**	1					
Bax	-.832**	-.850**	-.875**	1				
BCl2	.889**	.871**	.904**	-.875**	1			
EGFR	.918**	.900**	.861**	-.839**	.921**	1		
H1F1	.895**	.927**	.880**	-.891**	.877**	.931**	1	
VEGF	.896**	.896**	.879**	-.918**	.936**	.879**	.866**	1
N = 15								
** Correlation is significant at the 0.01 level (2-tailed).								

References:

1. Hudlikar, R.R., et al., *Polymeric black tea polyphenols (PBPs) inhibit benzo(a)pyrene and 4-(methylnitrosamino)-1-(3-pyridyl)-1- butanone-induced lung carcinogenesis potentially through down-regulation of p38 and Akt phosphorylation in A/J mice.* Mol Carcinog, 2017. **56**(2): p. 625-640.
2. Maru, G., *Isolation and analyses of polymeric polyphenol fractions from Black tea.* Food Chemistry - FOOD CHEM, 2006. **94**: p. 331-340.
3. Garg, R., A. Ingle, and G. Maru, *Dietary turmeric modulates DMBA-induced p21ras, MAP kinases and AP-1/NF-kappaB pathway to alter cellular responses during hamster buccal pouch carcinogenesis.* Toxicol Appl Pharmacol, 2008. **232**(3): p. 428-39.
4. Yapijakis, C., et al., *The Hamster Model of Sequential Oral Carcinogenesis: An Update.* In Vivo, 2019. **33**(6): p. 1751-1755.

Supplementary Figure:

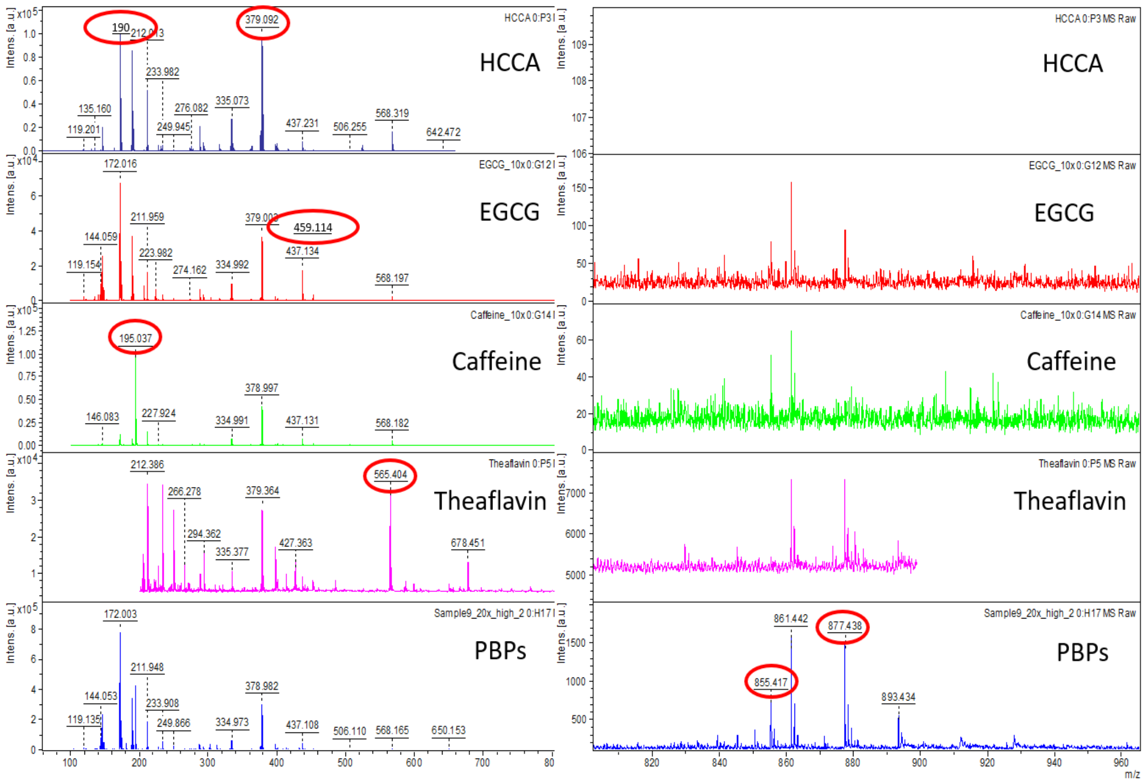

Supplementary figure 1 (SF1): Analysis of black tea derived PBPs by MALDI-TOF to evaluate black tea derived contaminants as EGCG, Caffeine and theaflavins. Representative MALDI-TOF spectra with a-cyano-4-hydroxycinnamic acid (HCCA) as a matrix, shows EGCG (m/z 459.11), caffeine (m/z 195.03) and theaflavin (m/z 565.40) specific peaks. Black tea derived PBP sample spectra were free from any of these contaminant signals which confirmed their purity, Further, PBPs showed presence of two PBP specific peaks with m/z values 855.41 and 877.43.

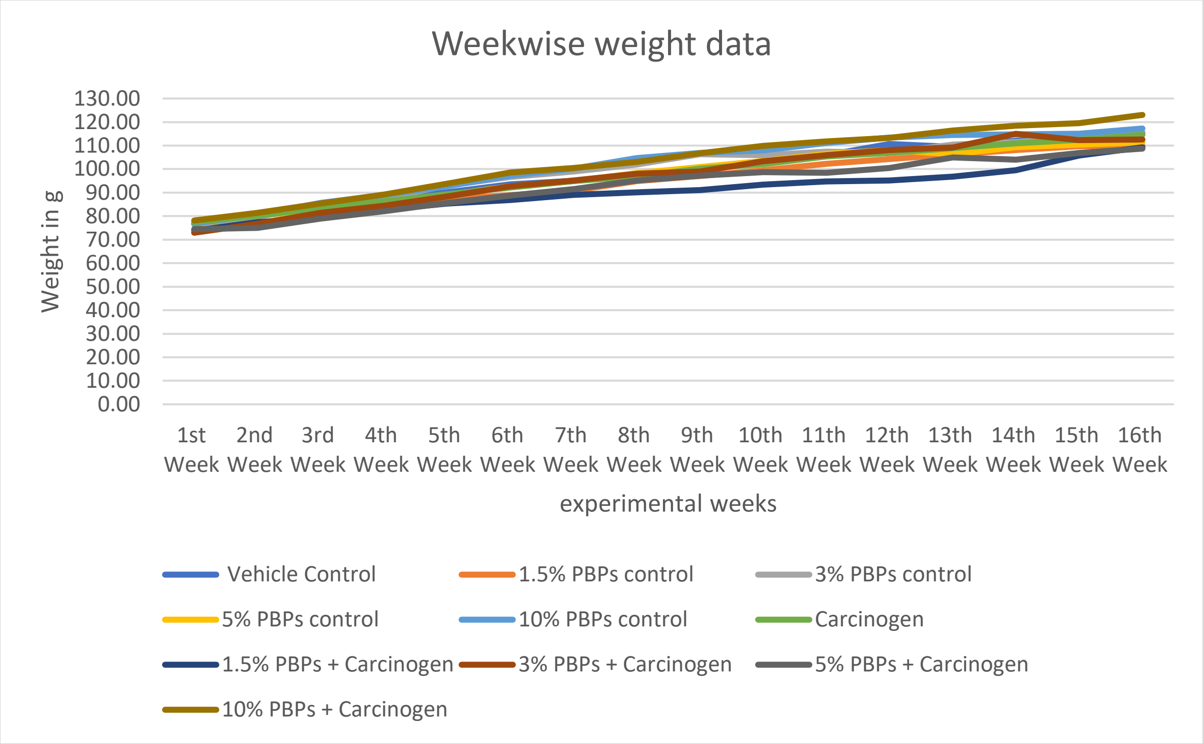

The data is average of vehicle control (n=31), 1.5% PBPs control (n=18), 3% PBPs control (n=13), 5% PBPs control (n=14), 10% PBPs control (n=14), carcinogen (n=37), 1.5% PBPs +carcinogen (n=19), 3% PBPs +carcinogen (n=21), 5% PBPs + carcinogen (n=22), 10% PBPs +carcinogen (n=22) animals.

Supplementary figure 2 (SF2): Week-wise data of animal weights in entire experimental period. A consistent increase in weights of all animals irrespective of the experimental group is observed.

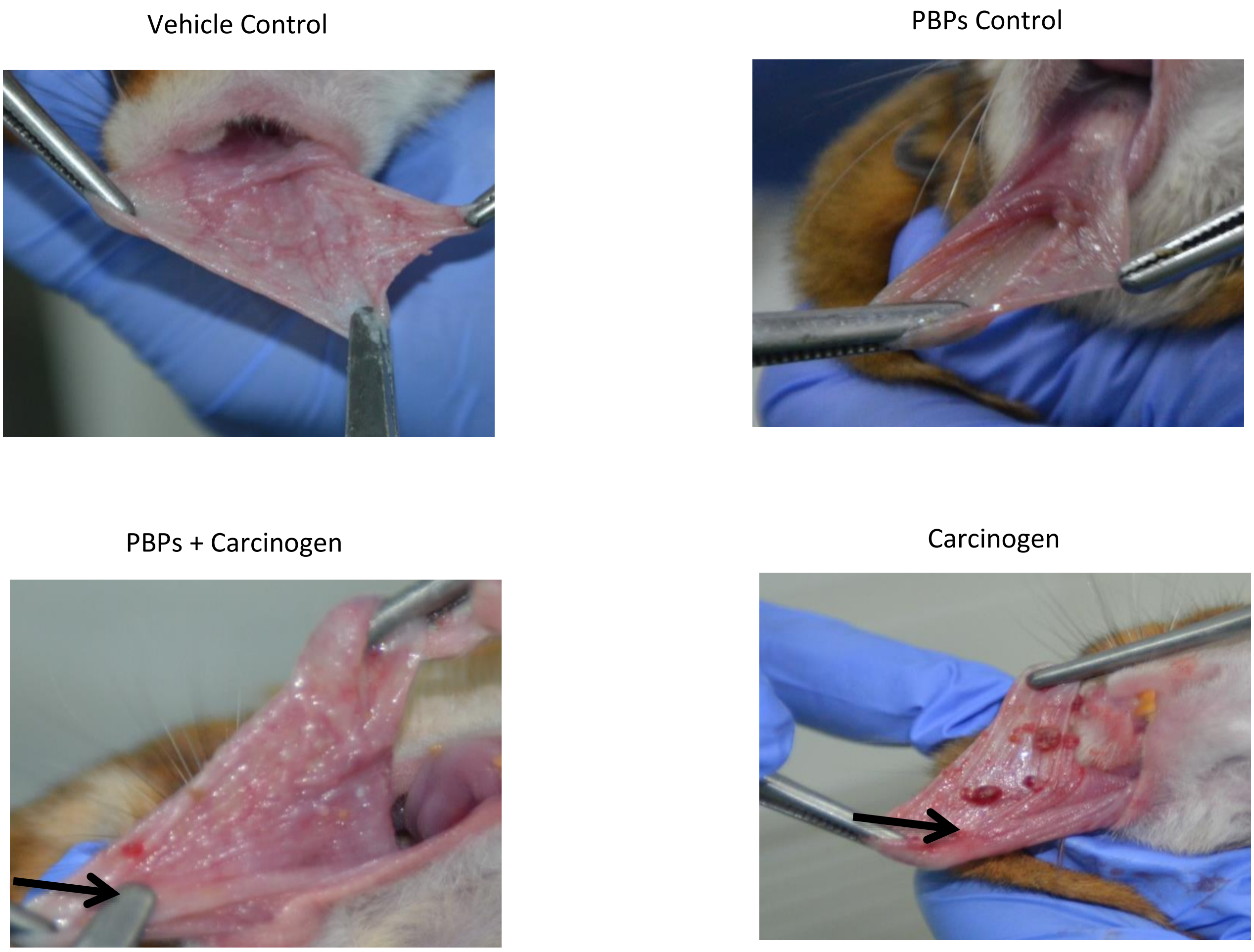

Supplementary figure 3 (SF3): Gross tumor appearance of DMBA induced hamster buccal pouch tumors after 16 weeks of total carcinogen and PBPs treatment in different treatment groups.

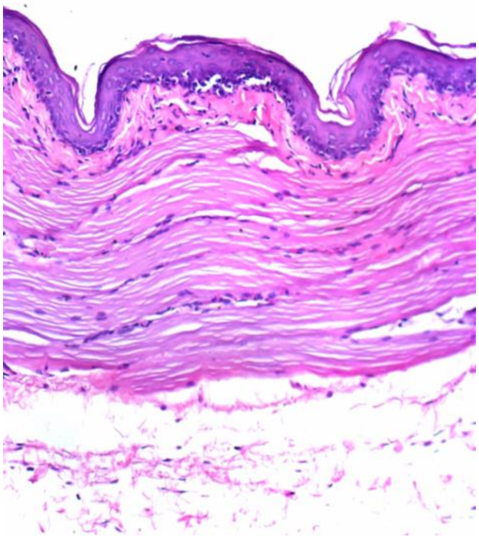

Normal Epithelium

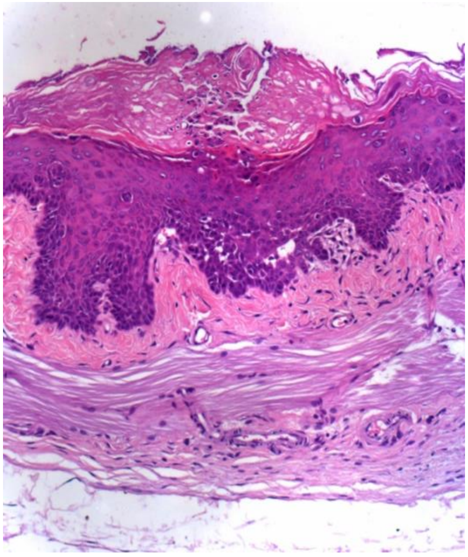

Hyperplasia

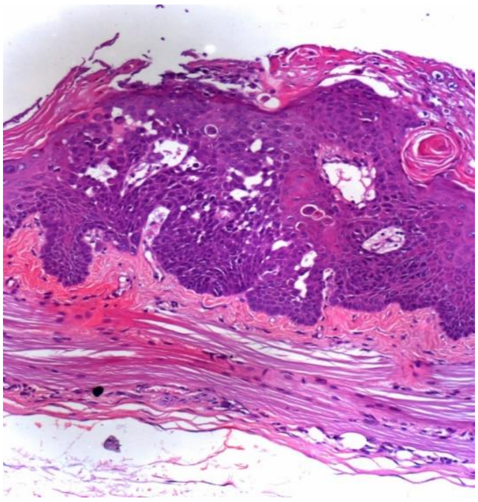

Dysplasia

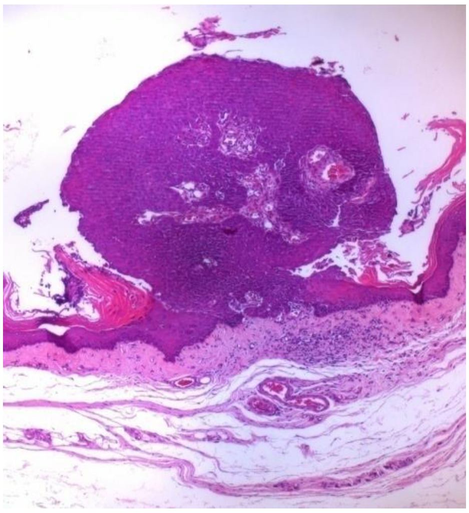

Squamous cell carcinoma

Supplementary figure 4 (SF4): Haematoxylin and eosin staining of hamster buccal pouch tissue demonstrating sequential progression of disease

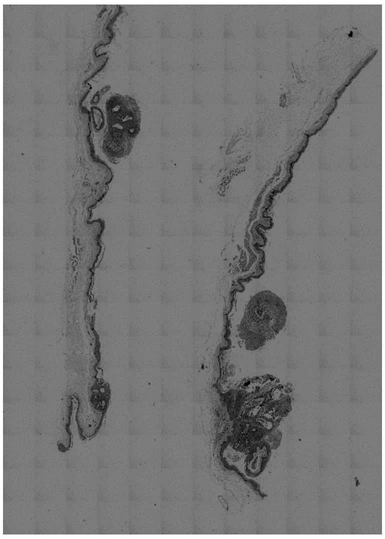

Carcinogen

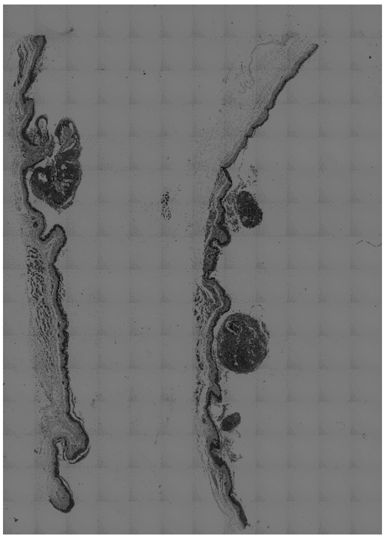

1.5% P+ C

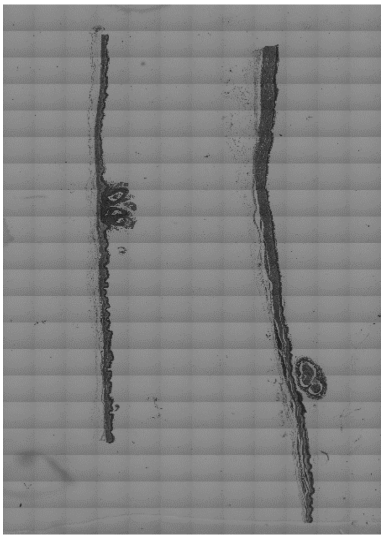

5% P+ C

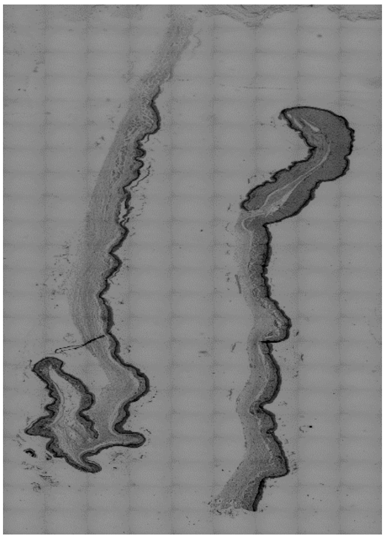

10% P+ C

Supplementary figure 5 (SF5): Representative tile scan images showing microscopic hamster buccal pouch tumors in C, 1.5% P+ C, 5% P+ C and 10% P+ C groups after 14 weeks of carcinogen treatment (magnification total 100 X).

A.

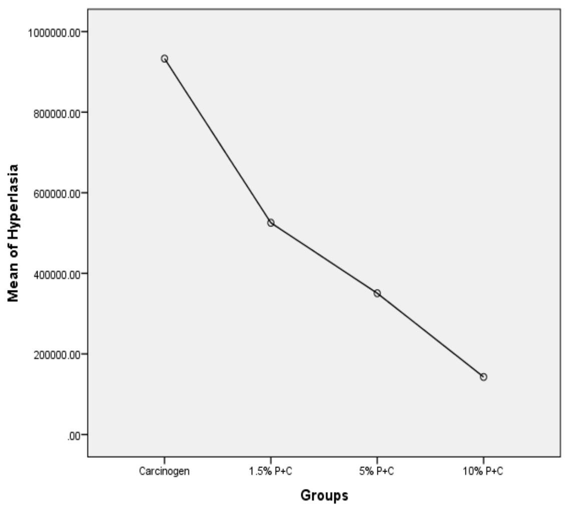

B.

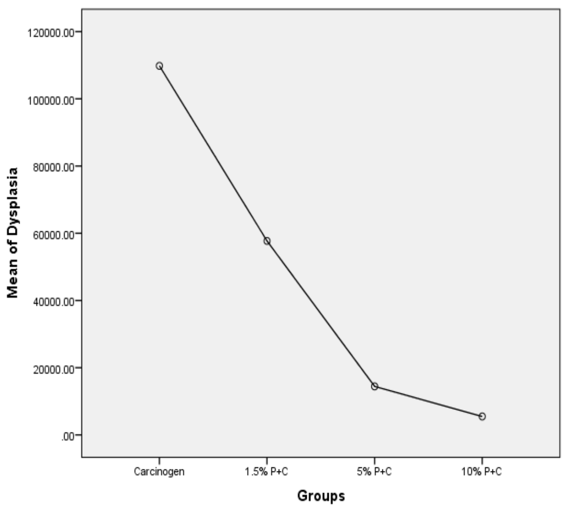

C.

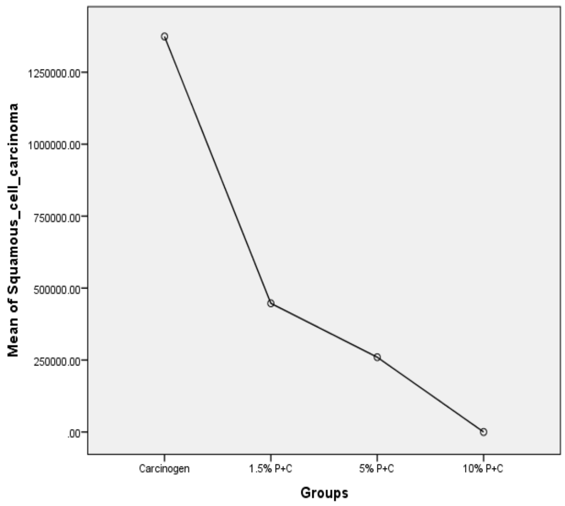

D.

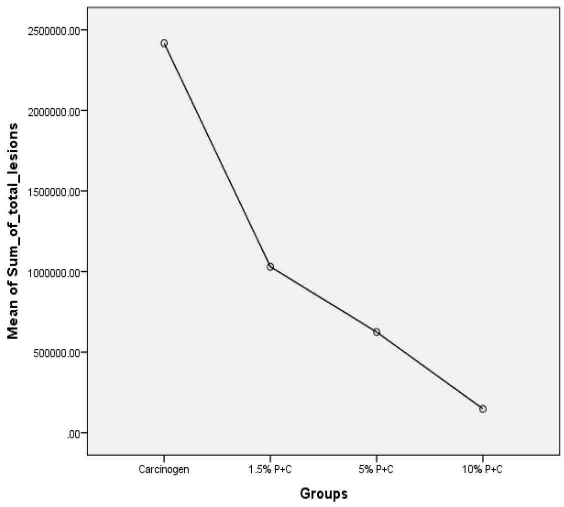

Supplementary figure 6 (SF6): Mean plots of effect of pre and concurrent treatment of PBPs on different microscopic hamster buccal pouch lesion areas as A. Hyperplasia, B. Dysplasia, C. Squamous cell carcinoma and D. Sum total of these lesions together

A.

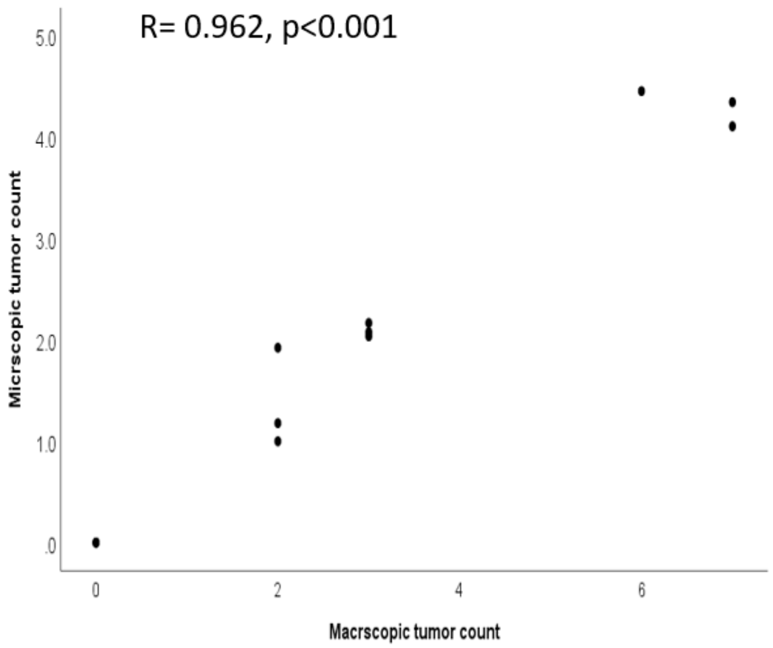

Supplementary figure 7 (SF7): Correlation analysis of macroscopic tumor count and microscopic tumor count.

A.

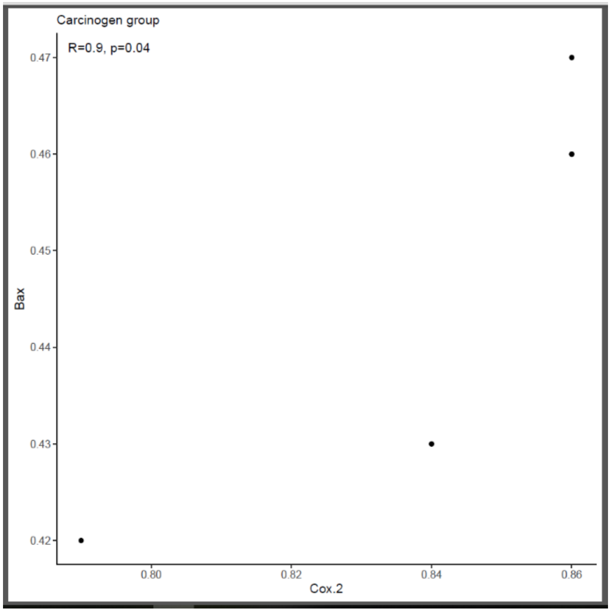

B.

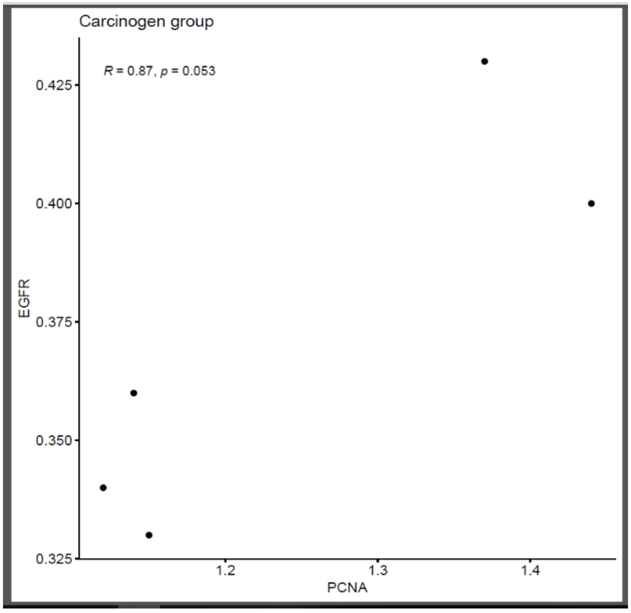

C.

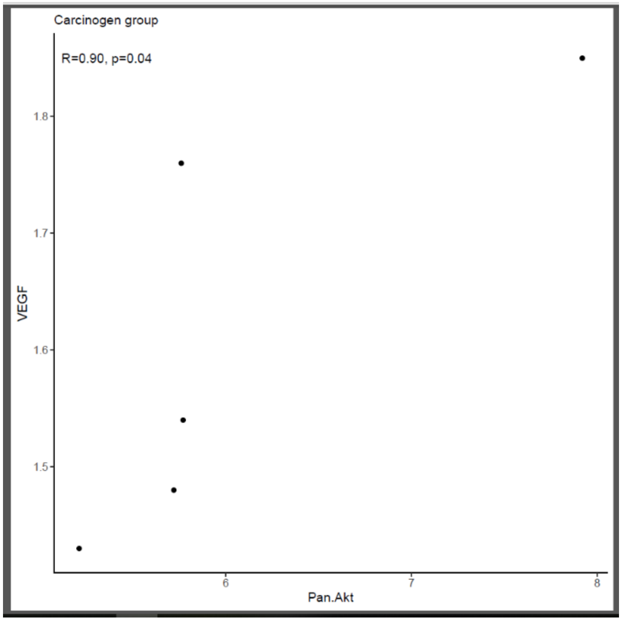

Supplementary figure 8 (SF8): Correlation analysis between different molecular markers in hamster buccal pouch tumor tissue **A.** Bax-Cox2 **B.** EGFR- PCNA and **C.** VEGF- pan Akt.

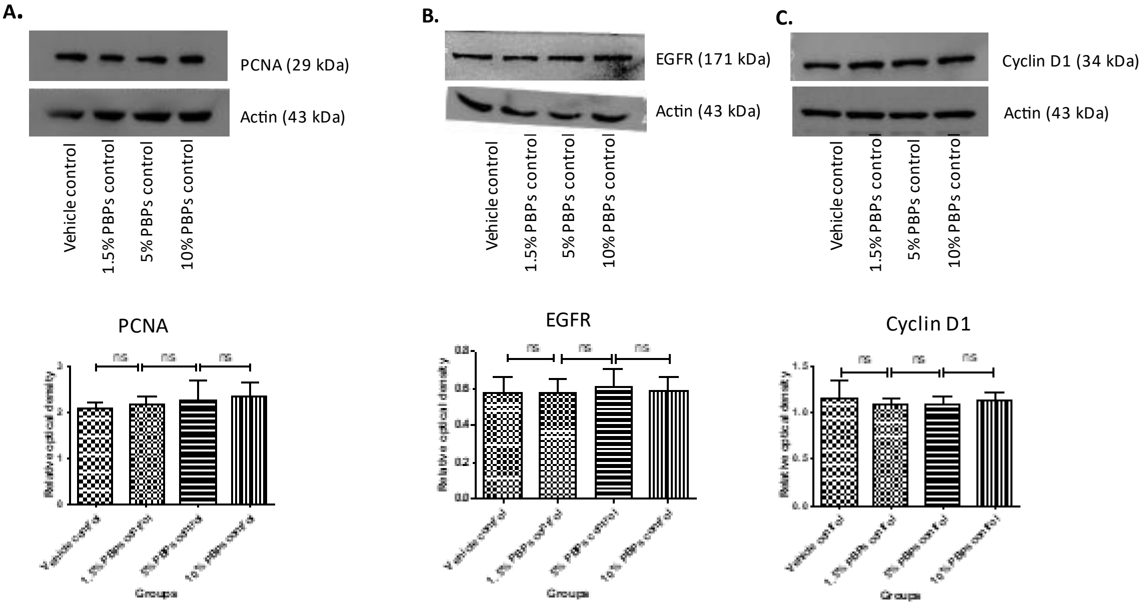

Data represented as mean ± S.D. of three observations. (ns, non significant ANOVA followed by Bonneferoni's correction).

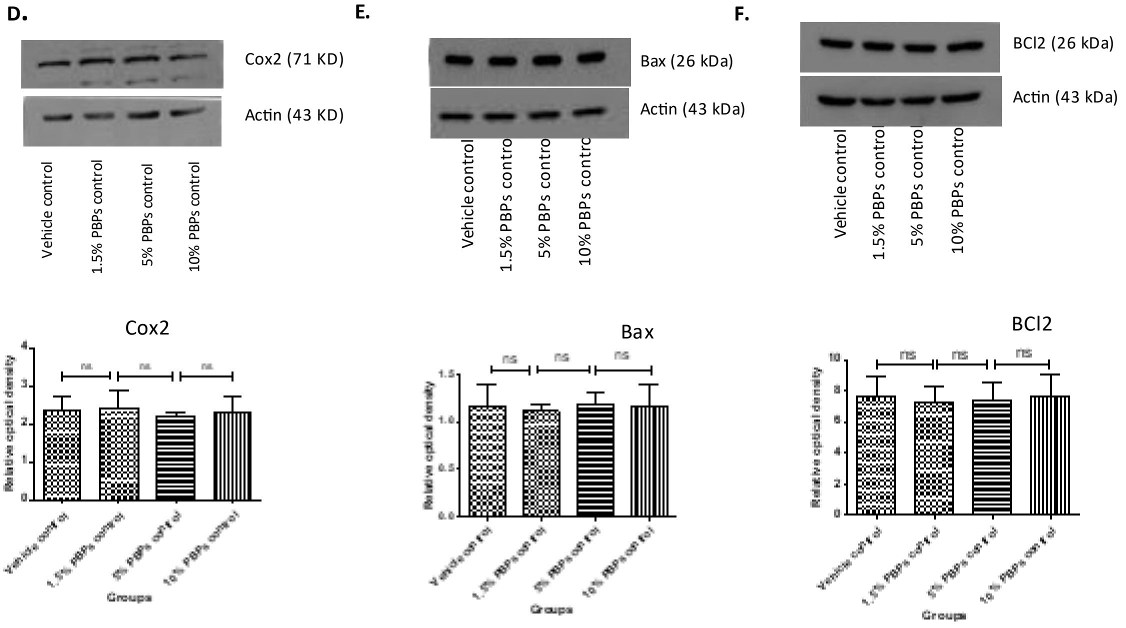

Data represented as mean ± S.D. of three observations. (***, p≤0.0001, ANOVA followed by Bonneferoni's correction).

G.

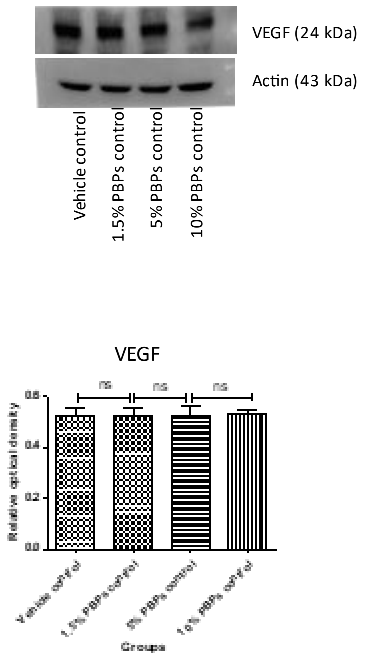
VEGF (24 kDa)
Actin (43 kDa)
Vehicle control
1.5% PBPs control
5% PBPs control
10% PBPs control
VEGF
ns
Relative optical density
Groups

H.

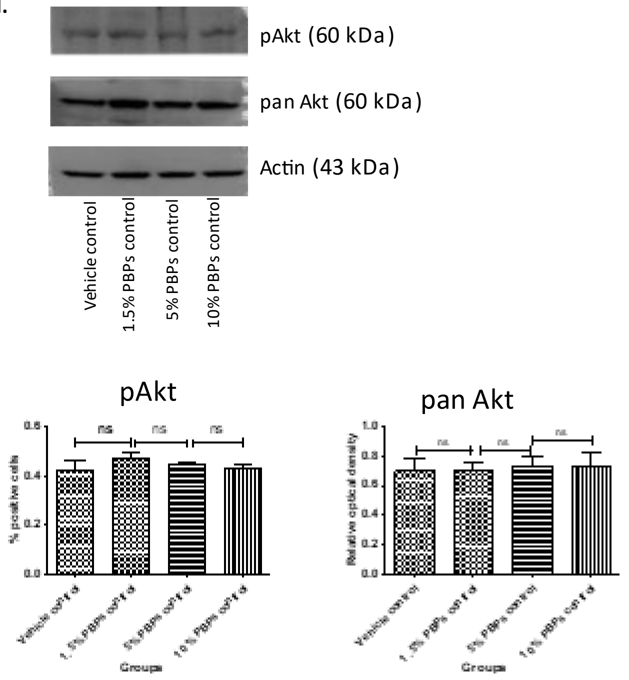
pAkt (60 kDa)
pan Akt (60 kDa)
Actin (43 kDa)
Vehicle control
1.5% PBPs control
5% PBPs control
10% PBPs control
pAkt
ns
% positive cells
Groups
pan Akt
Relative optical density
Groups

I.

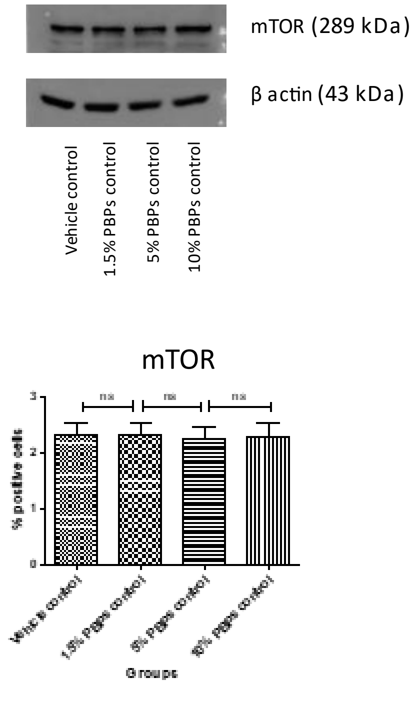
mTOR (289 kDa)
β actin (43 kDa)
Vehicle control
1.5% PBPs control
5% PBPs control
10% PBPs control
mTOR
ns
% positive cells
Groups

Supplementary figure 9 (SF9): Expression of A. PCNA, B. EGFR, C. Cyclin D1, D. Cox2, E. Bax and F. BCl2 G. VEGF, H. pAkt and Akt I. mTOR across all the control groups i.e., Vehicle control, 1.5% PBPs control, 5% PBPs control and 10% PBPs control.

Whole Raw Blots:

Original supplementary figure 9 D.

COX2- control

COX-2

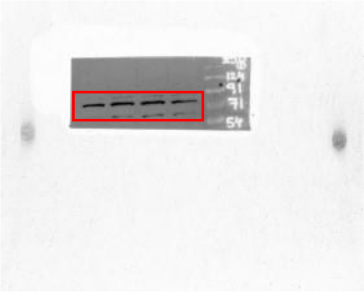

Actin

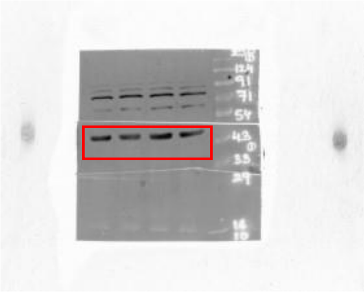

Original Figure 2 A. a.

COX2

COX-2

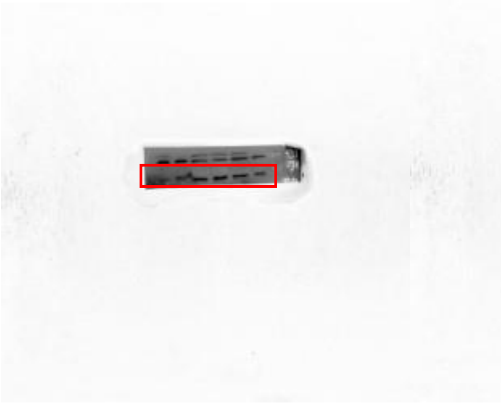

Actin

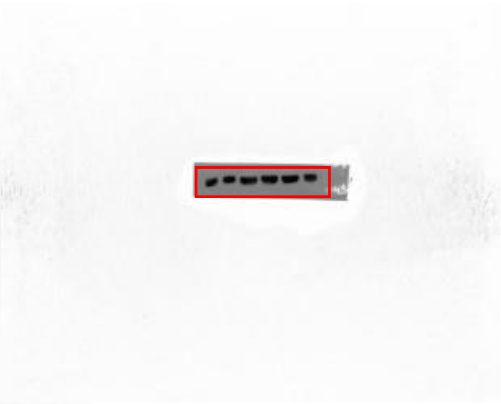

All replicates for Cox2

Test

Control

Actin

Cox2

Actin

Cox2

Original supplementary figure 9 D.

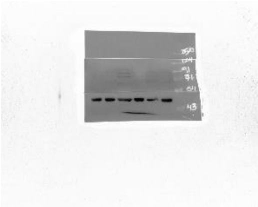

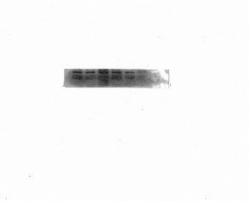

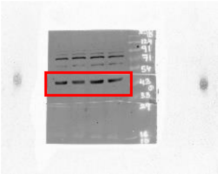

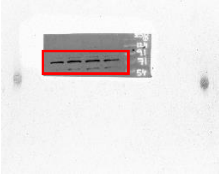

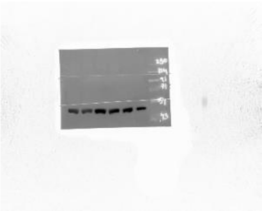

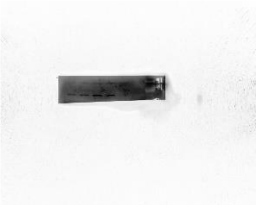

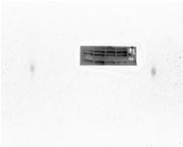

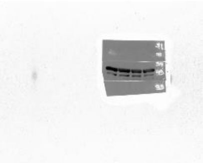

Original Figure 2 A. a.

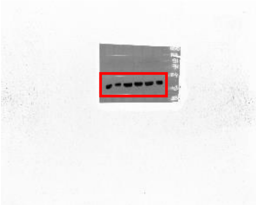

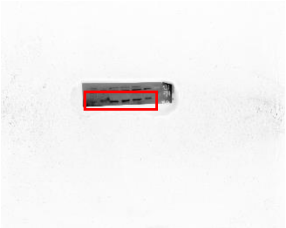

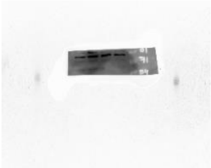

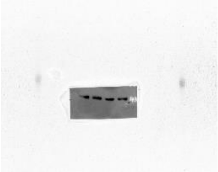

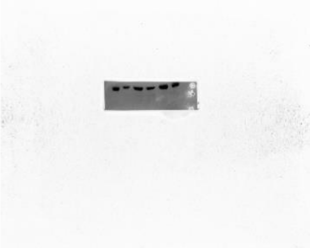

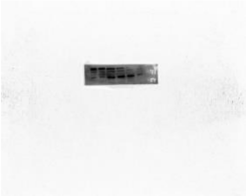

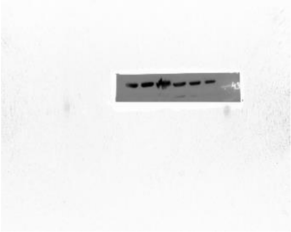

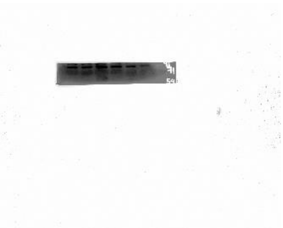

Original supplementary figure 9 A.

PCNA - control

PCNA

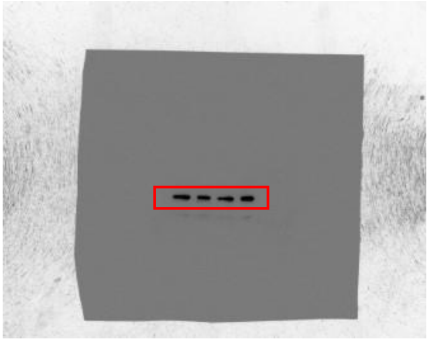

Actin

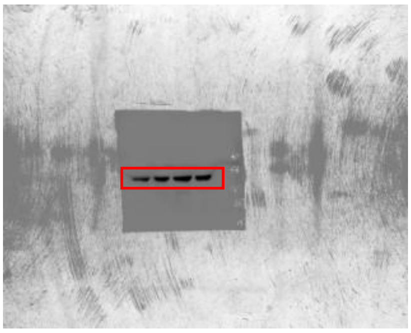

Original Figure 2 A. b.

PCNA

PCNA

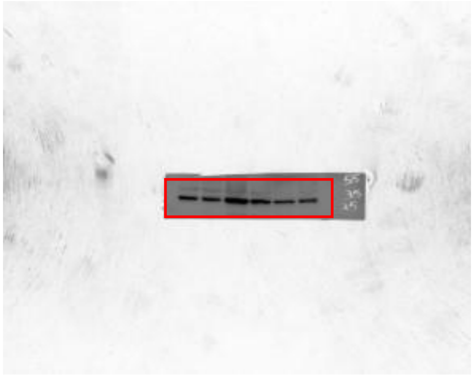

Actin

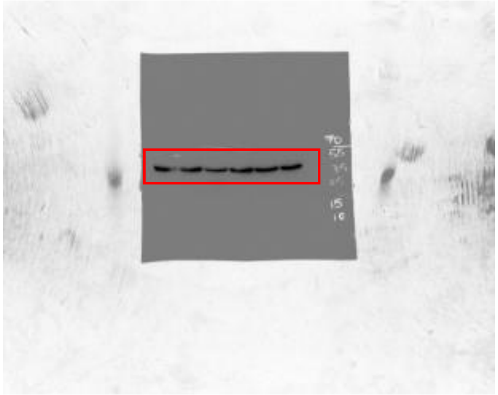

All replicates for PCNA

Test

Control

Actin

PCNA

Actin

PCNA

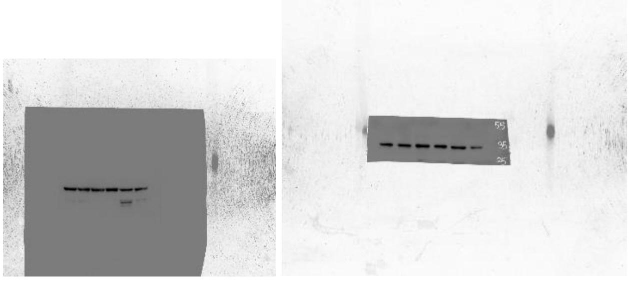

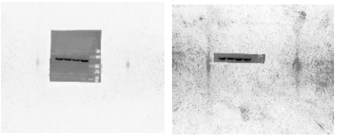

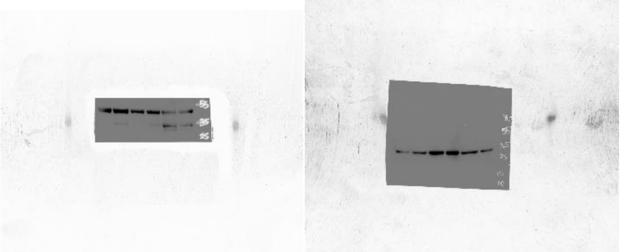

Original supplementary figure 9 A.

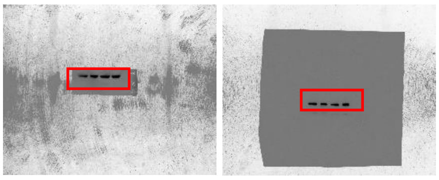

Original Figure 2 A. b.

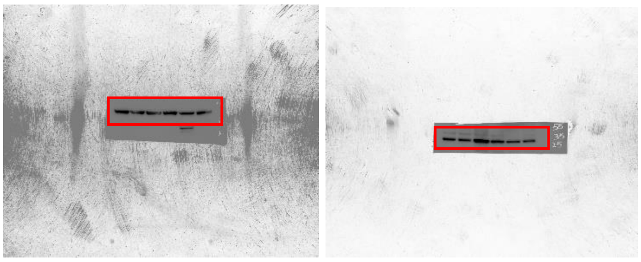

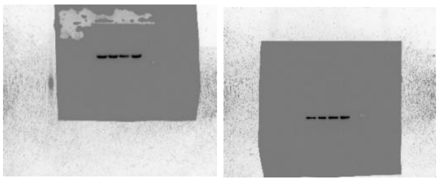

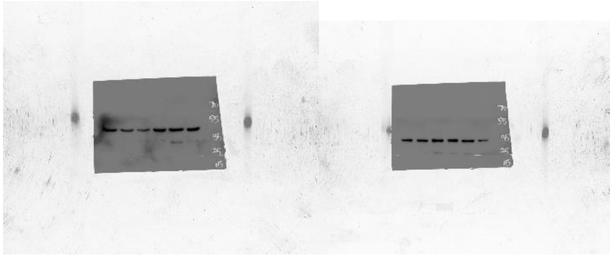

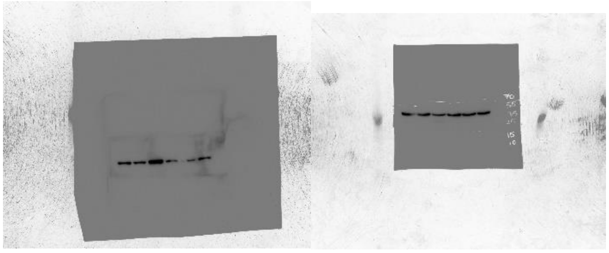

Original Supplementary figure 9. E.

Bax - control

Bax

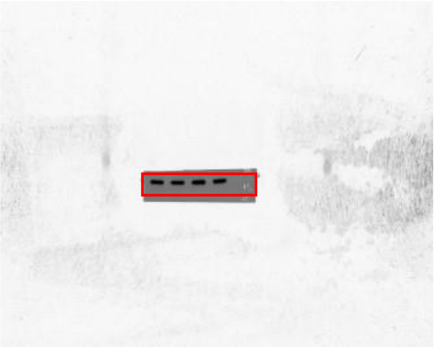

Actin

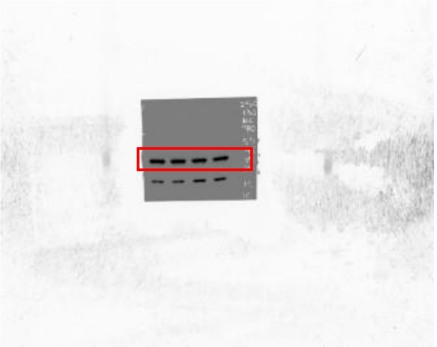

Original Figure 2 A. c.

Bax

Bax

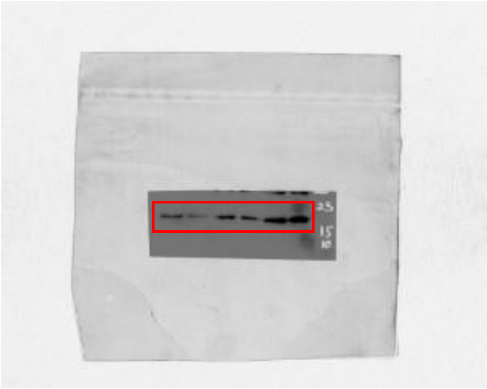

Actin

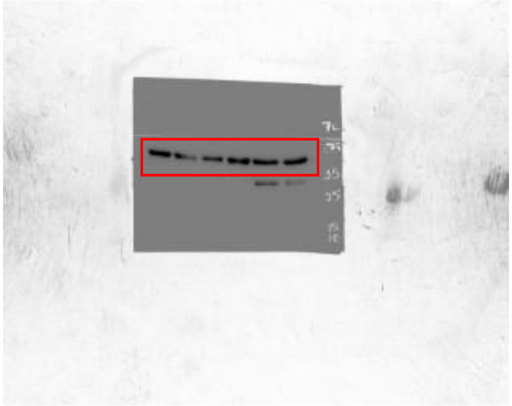

All replicates for Bax

Test

Actin Bax

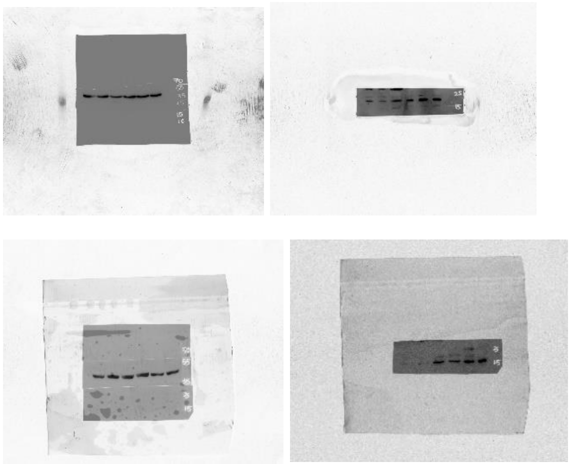

Control

Actin Bax

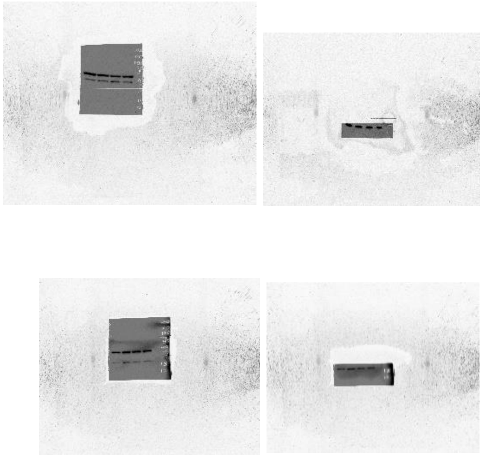

Original Figure 2 A. c.

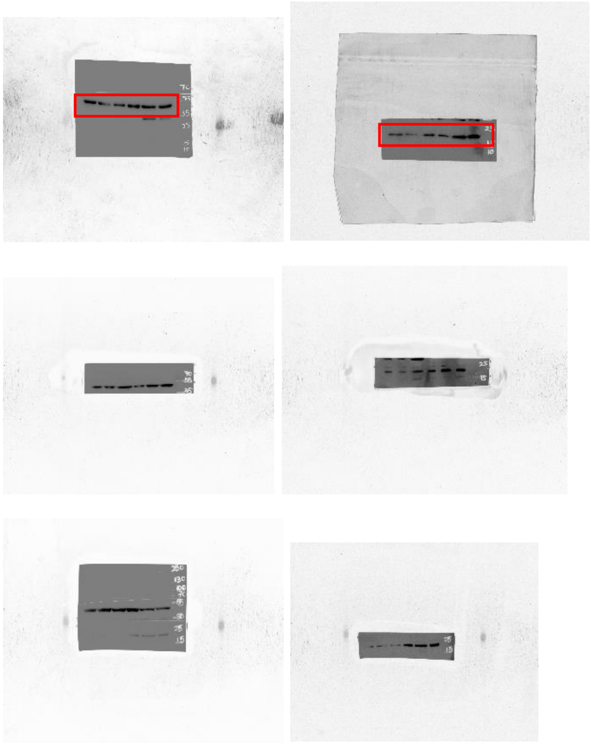

Original Supplementary figure 9. E.

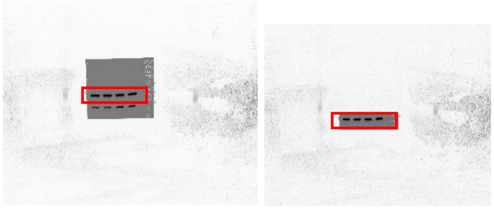

Original Supplementary figure 9. F.

Bcl2 - control

Bcl2

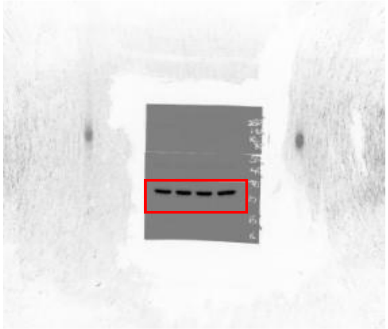

Actin

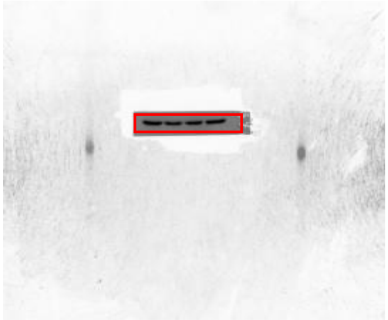

Original Figure 2 A. d.

Bcl2

Bcl2

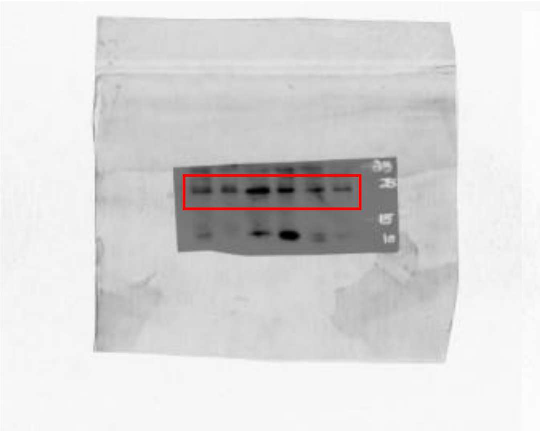

Actin

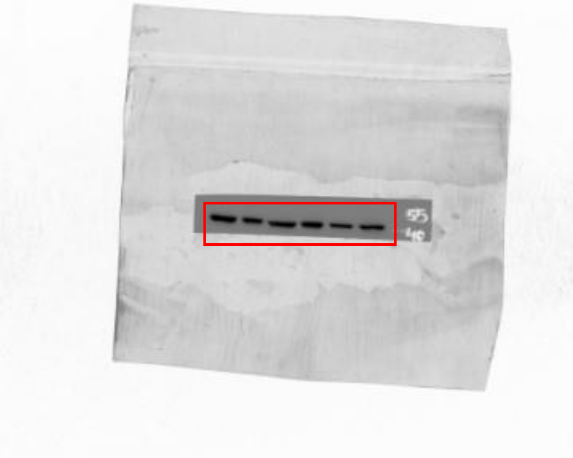

All replicates for Bcl2

Test

Control

Actin

Bcl2

Actin

Bcl2

Original Figure 2 A. d.

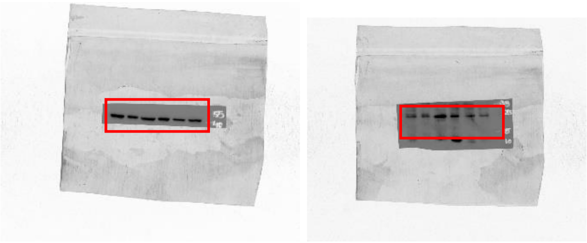

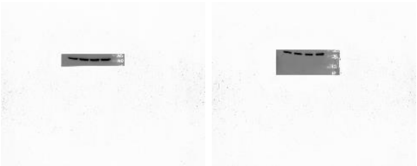

Original Supplementary figure 9. F.

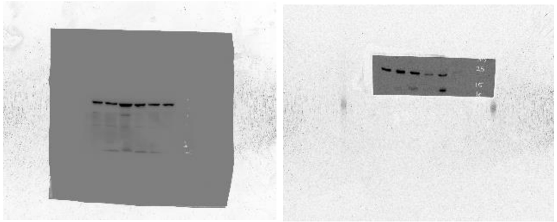

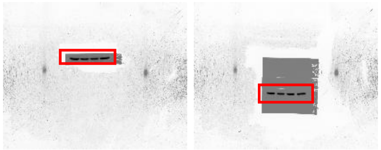

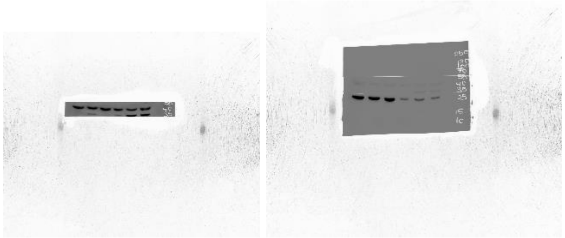

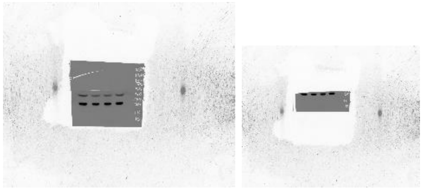

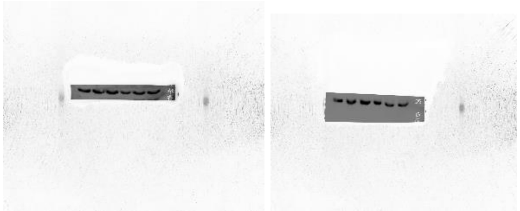

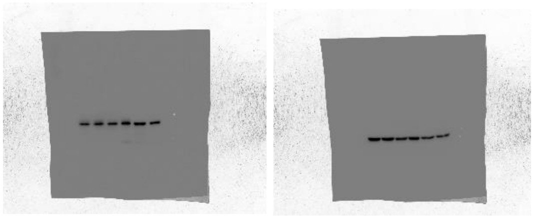

Original Supplementary figure 9 B.

EGFR- control

EGFR

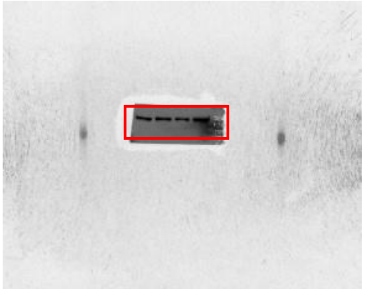

Actin

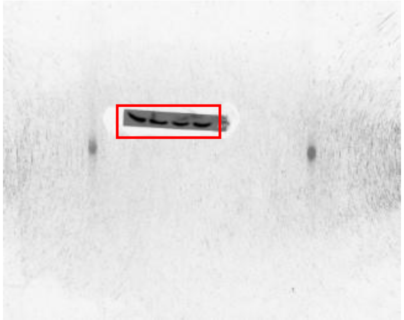

Original Figure 3. A. a.

EGFR

EGFR

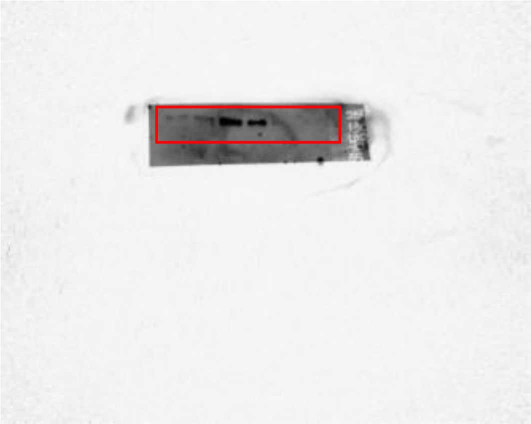

Actin

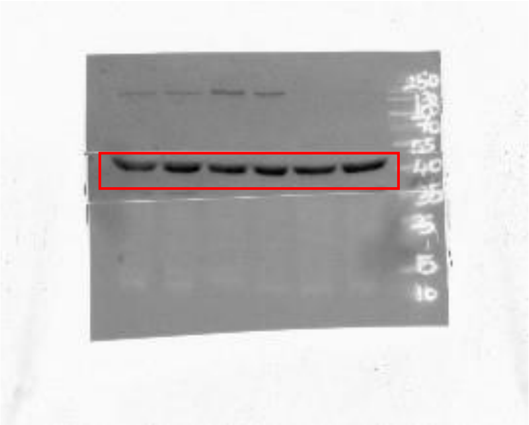

All replicates for EGFR

Test

Actin EGFR

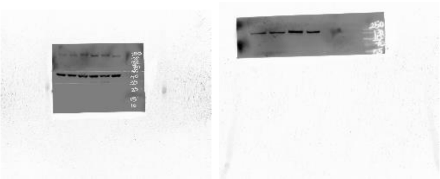

Original Figure 3. A. a.

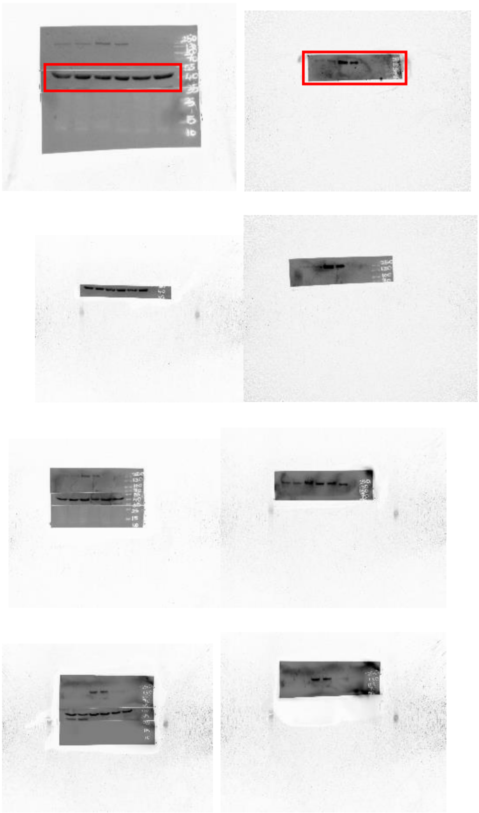

Control

Actin EGFR

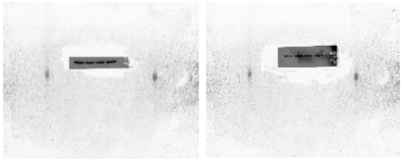

Original Supplementary figure 9 B.

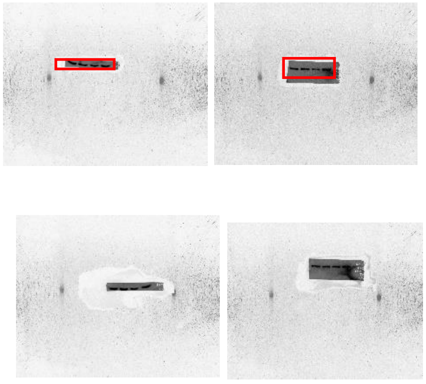

Original Supplementary figure 9 C.

Cyclin D1- control

CyclinD1

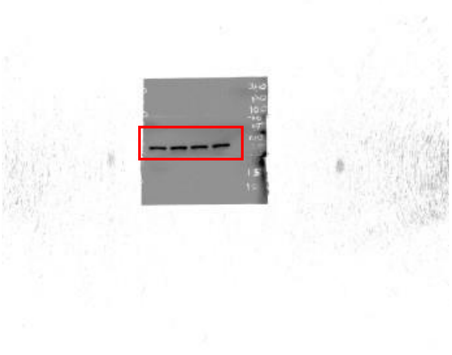

Actin

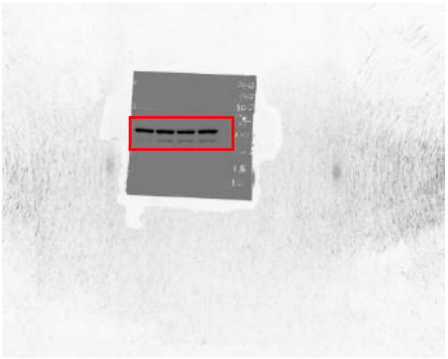

Original Figure 3 A. b.

Cyclin D1

CyclinD1

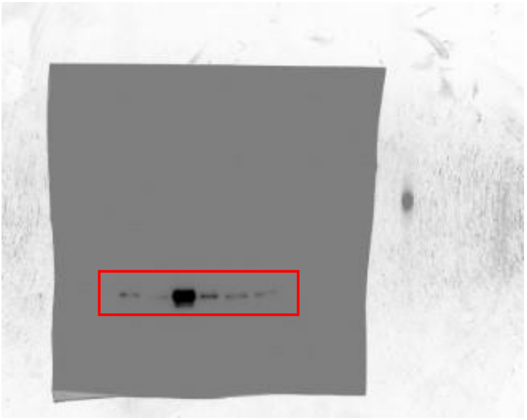

Actin

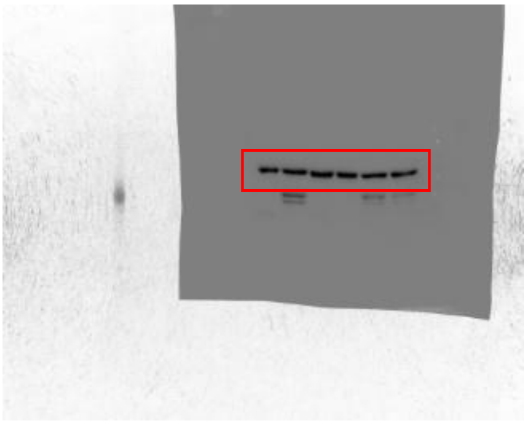

All replicates for CyclinD1

Test Control

Actin CyclinD1 Actin CyclinD1

Original Figure 3 A. b.

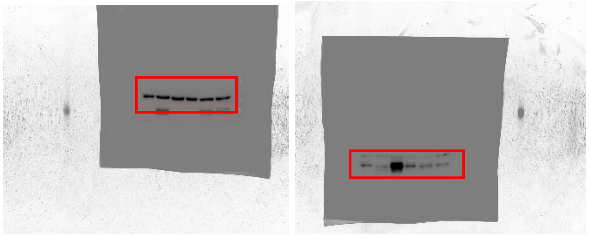

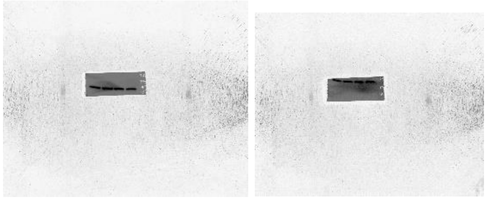

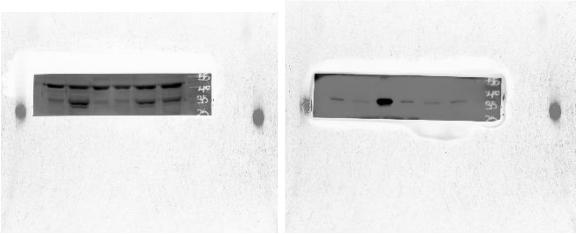

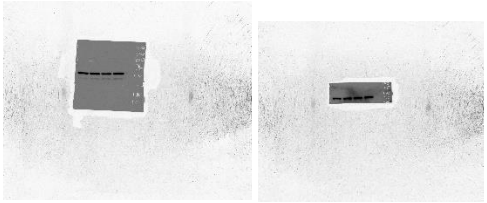

Original supplementary figure 9 C.

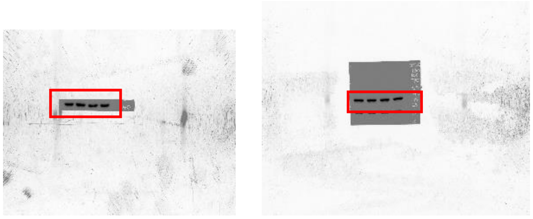

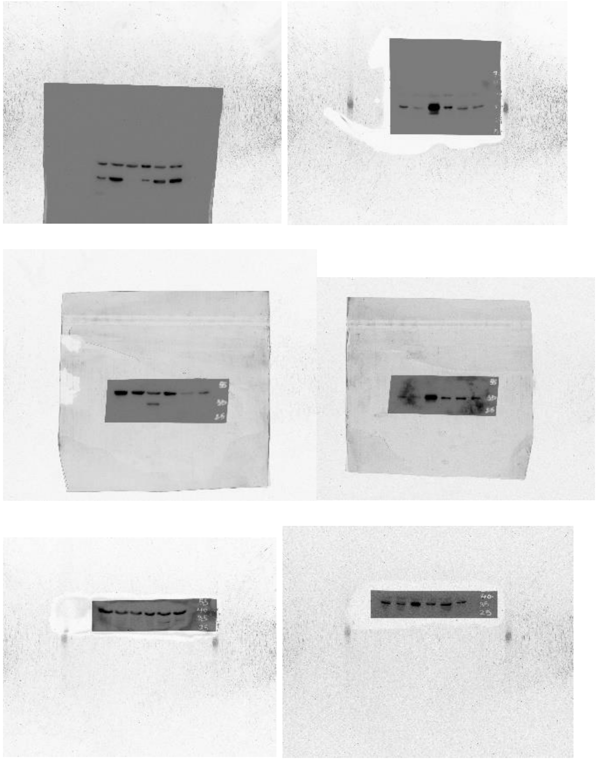

Original supplementary figure 9 H.

pAkt- control

pAkt Actin

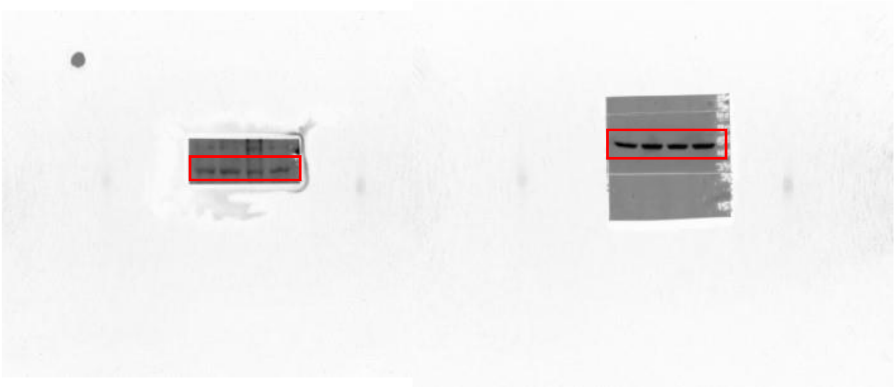

Original Figure 3. A. c.

pAkt

pAkt Actin

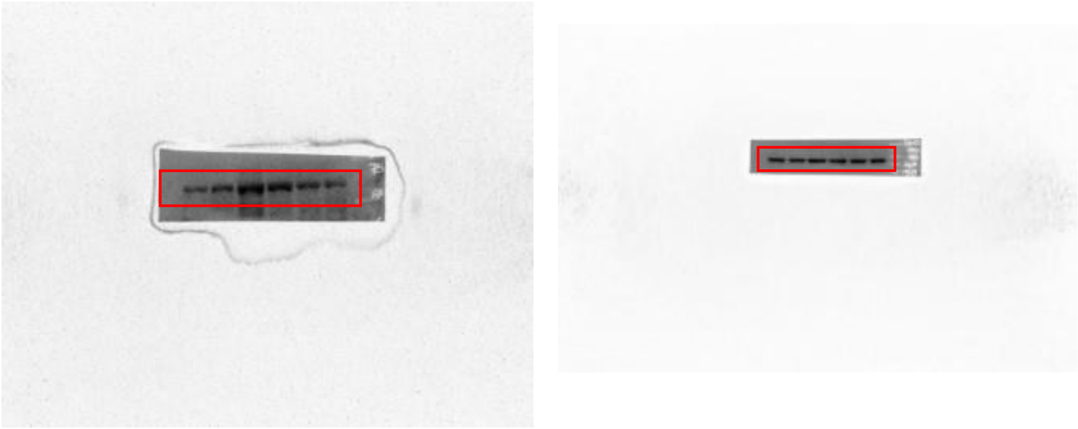

All replicates for pAkt

Test

Actin pAkt

Original Figure 3. A. c.

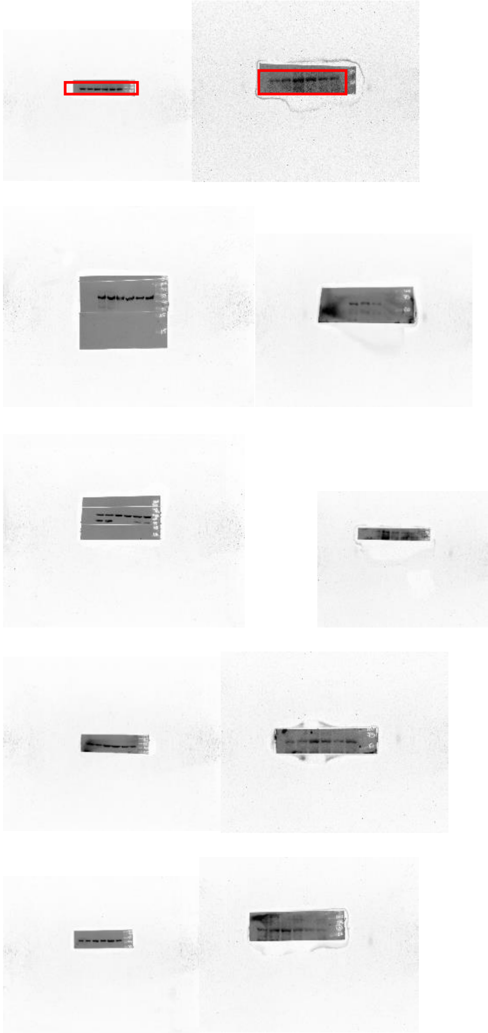

Control

Actin pAkt

Original supplementary figure 9 H.

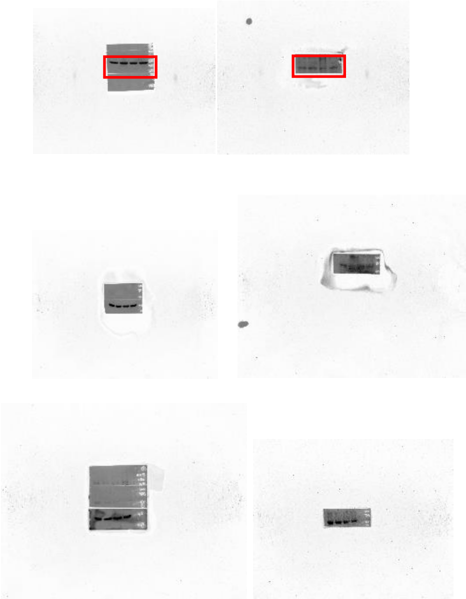

Original Supplementary figure 9. H.

Akt- control

Akt

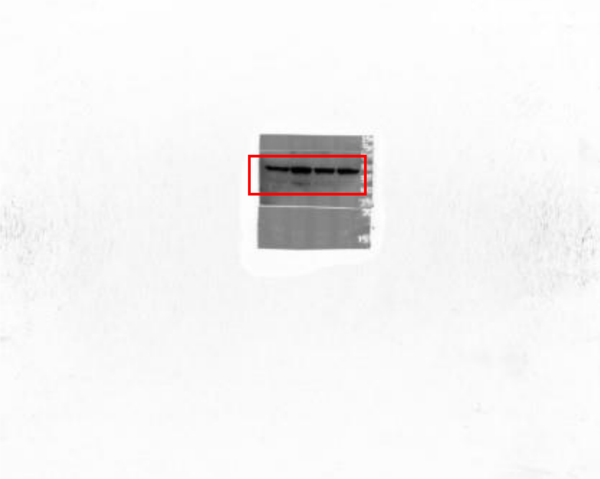

Actin

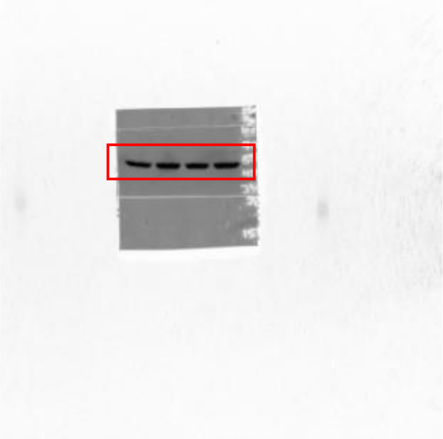

Original Figure 3 A. d.

Akt

Akt

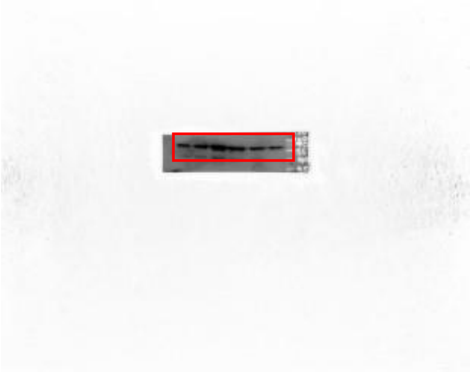

Actin

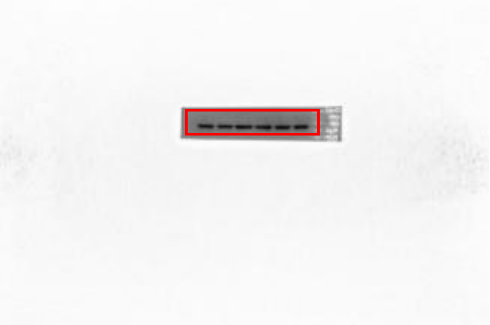

All replicates for Akt

Test Control

Actin Akt Actin Akt

Original Figure 3 A. d.

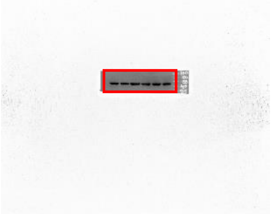
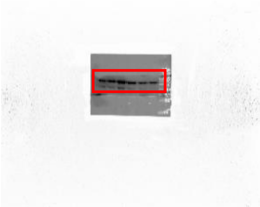
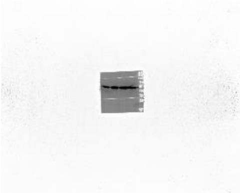
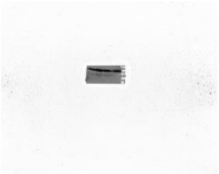

Original Supplementary figure 9. H.

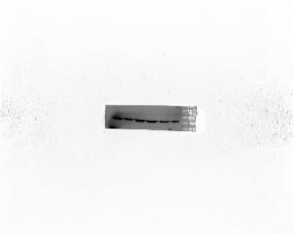
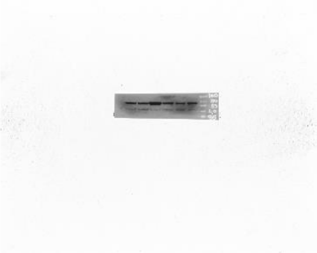
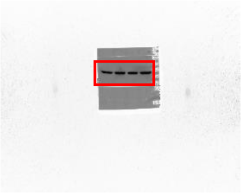
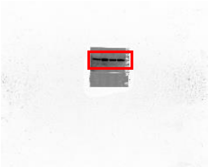
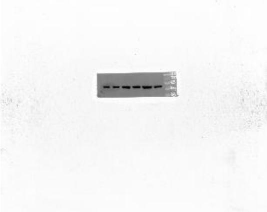
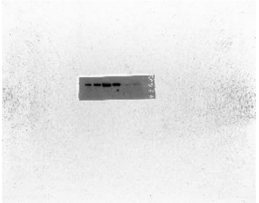
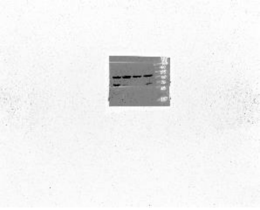
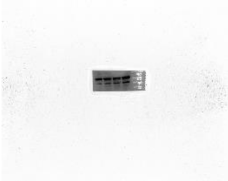
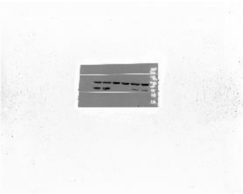
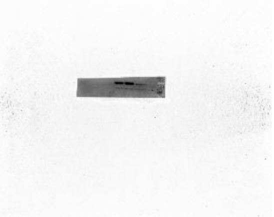
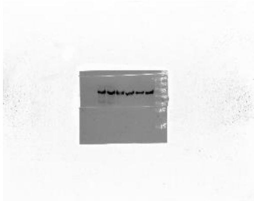
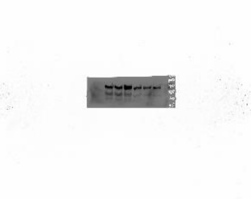

Original Supplementary figure 9. I.

mTOR- control

mTOR

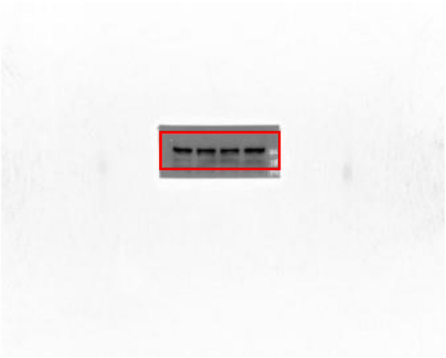

Actin

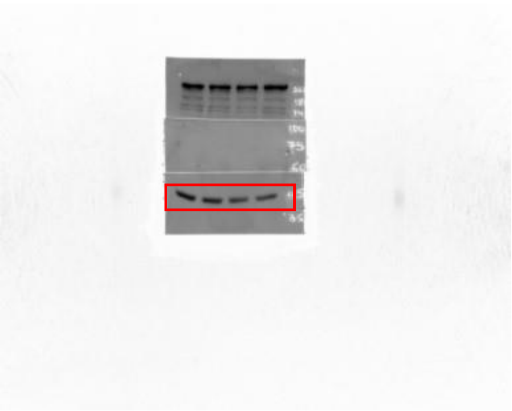

Original Figure 3 A. e.

mTOR

mTOR

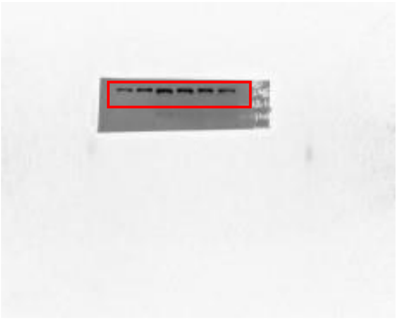

Actin

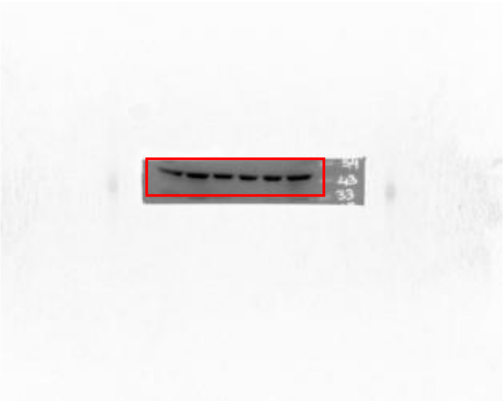

All replicates for mTOR

Test

Control

Actin

mTOR

Actin

mTOR

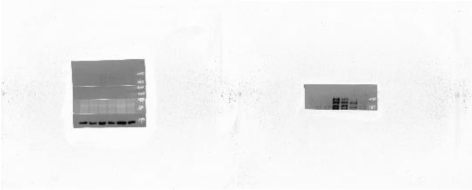

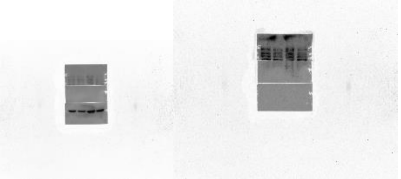

Original supplementary figure 9. I.

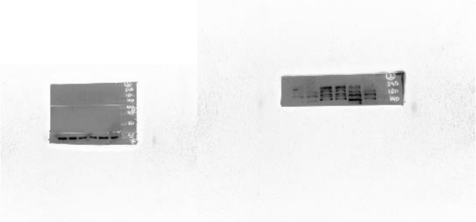

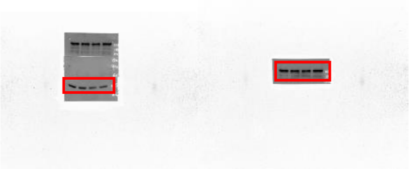

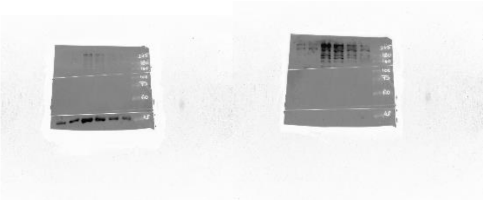

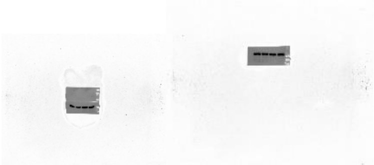

Original Figure 3 A. e.

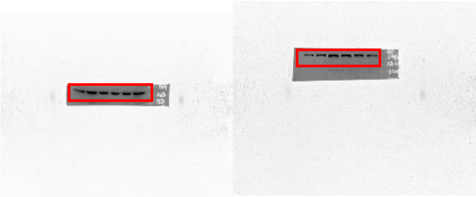

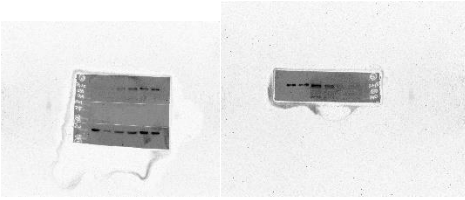

Original Supplementary figure 9 G.

VEGF- control

VEGF Actin

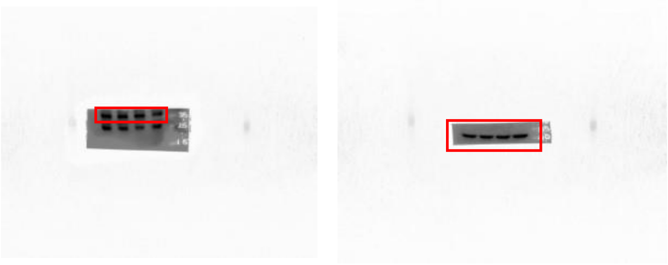

Original Figure 4. B.

VEGF

VEGF Actin

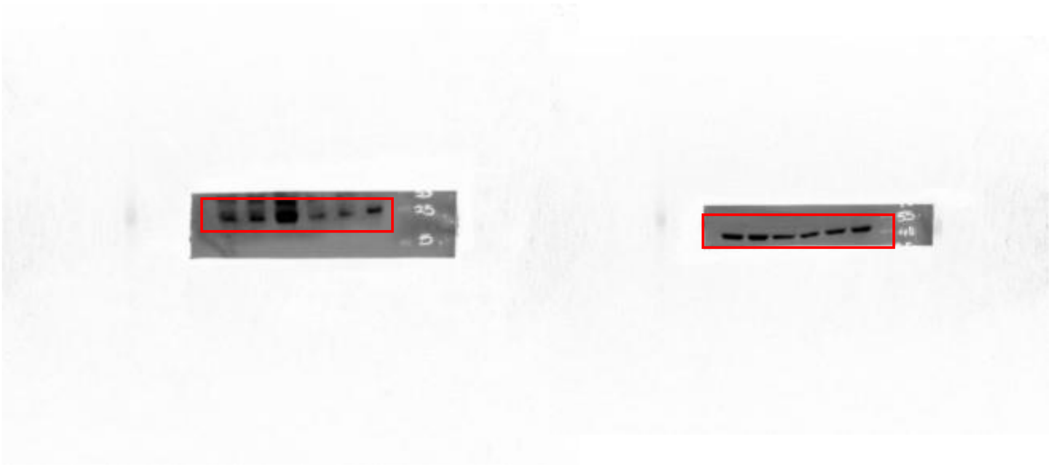

All replicates for VEGF

Test

Actin

VEGF

Control

Actin

VEGF

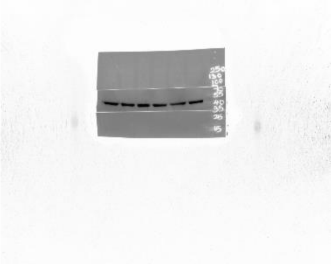

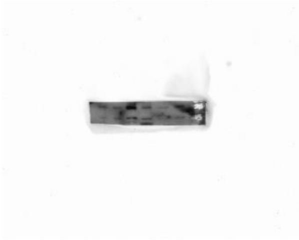

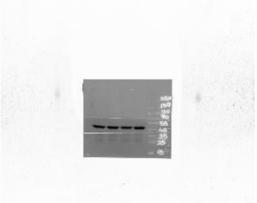

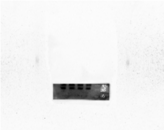

Original Supplementary figure 9 G.

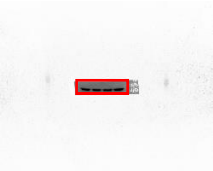

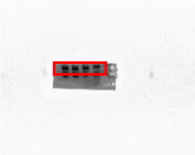

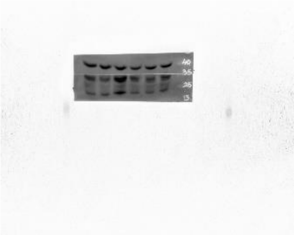

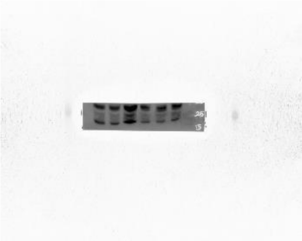

Original Figure 4. B.

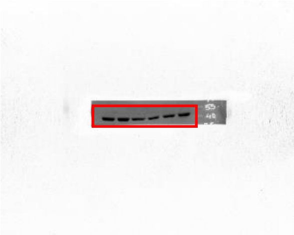

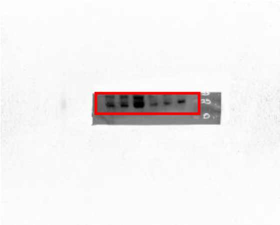

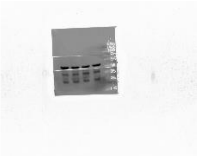

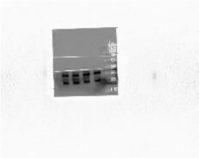

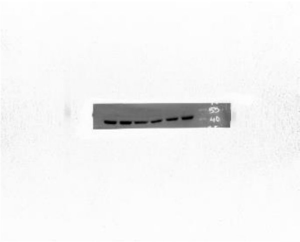

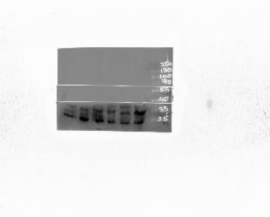

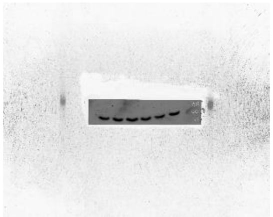

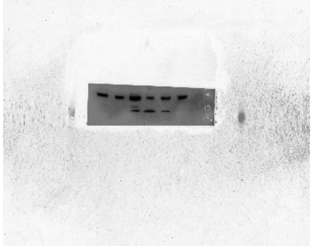

Supplement: Supplementary file 1 — Supplementary Information. [file 41598_2022_18680_MOESM1_ESM.pdf]
